# Welcome to the house-warming party: social interactions and antagonism against *Listeria monocytogenes* in multi-species biofilms

**DOI:** 10.1128/aem.01386-26

**Published:** 2026-09-30

**Authors:** Yatong C. Jiang, Ranine El-Jurdi, Yuen Ying Chan, Donghe M. Deng, Zhaohui S. Xu, Michael G. Gänzle

**Affiliations:** 1 Department of Agricultural, Food and Nutritional Science, University of Alberta 461698, Edmonton, Canada; Indiana University Bloomington, Bloomington, Indiana, USA

**Keywords:** *Listeria monocytogenes*, biofilm, bacteriocins, quorum sensing, *Carnobacterium*

## Abstract

*Listeria monocytogenes* integrates into multi-species biofilms and persists in food processing facilities. Its persistence depends on the composition of the microbial communities, but those species that accommodate *L. monocytogenes*, or not, remain to be identified. Moreover, the decision-making on biofilm vs pellicle formation in microbial communities remains poorly understood. It was the aim of this study to identify communities that accommodate *Listeria*, or not, and to observe biofilm vs pellicle formation in multi-species biofilms. We quantified *L. monocytogenes* in six reconstituted communities, each consisting of 5–19 strains. A 15-member community was simplified into five species to quantify *L. monocytogenes* in all 31 combinations of these five strains. *Janthinobacterium tructae* favored pellicle formation, while *Pseudomonas shahriarae* convinced others to join a surface-attached biofilm. In the six microbial communities, cell counts of *L. monocytogenes* were lowest when *Carnobacterium maltaromaticum* was also present. The antagonistic effect of *C. maltaromaticum* was confirmed when analyzing all combinations of five strains. The presence of *C. maltaromaticum* reduced cell counts of *L. monocytogenes* by about 1 log CFU/cm^2^. *Serratia liquefaciens* also antagonized *L. monocytogenes*, but this was observed (*P* < 0.05) only in the absence of *C. maltaromaticum*. Genome analyses of the community identified close to 100 different quorum-sensing systems, including peptides that are also bactericidal against *L. monocytogenes*. We conclude that the language used to describe human social interactions is also suited to describe microbial interactions in multi-species biofilm communities, but unraveling mechanisms of communication among biofilm-embedded bacteria remains subject to future studies.

**IMPORTANCE:**

Microbial communication in relation to biofilm vs pellicle formation and on whether to accommodate *Listeria monocytogene*s or not partially converge with quorum-sensing systems as a key mechanism. Two organisms, *Carnobacterium maltaromaticum* and *Serratia liquefaciens*, antagonized *L. monocytogenes*. Microbial interactions are strongly dependent on the complexity of the microbial community, and different behaviors were observed in biofilms formed by 2, 5, or 15 strains. Results presented in this study document that the use of protective cultures may also reduce persistence of *Listeria* in food processing facilities but are far from providing an understanding of communication and social rules in complex bacterial communities. The novel perspective on microbial interactions may direct future experiments.

## INTRODUCTION

*Listeria monocytogenes* is of concern for food safety due to the high mortality of listeriosis and the low infectious dose of *L. monocytogenes* in susceptible populations (1, 2). Listeriosis outbreaks have increased since 2000 (3), which links to the growing demand for ready-to-eat foods that support the growth of *L. monocytogenes* (4). *L. monocytogenes* persists in food processing facilities despite regular cleaning and sanitation (5, 6). A substantial proportion of food processing facilities harbor persistent bacterial communities that include *L. monocytogenes* (7).

Biofilm formation is the central mechanism enabling long-term microbial persistence. The biofilm matrix allows the community to remain in place despite rapid fluid flow and protects microbes from environmental stressors (8, 9). Biofilms are formed by one or more bacterial species embedded in a matrix of extracellular polymeric substances (10). Biofilms, referred to as “cities of microbes,” exhibit complex morphologies that are determined by social interactions among diverse inhabitants (11). The extracellular matrix (ECM) serves as a diffusion barrier and protects biofilm-embedded bacteria against sanitizers (12, 13). Biofilms are formed on biotic and abiotic surfaces (hereafter: biofilms) or at the air–liquid interface (hereafter: pellicles). Pellicles enable aerobic bacteria to access oxygen at the air–liquid interface (14, 15). Pellicles are observed in food fermentations such as kombucha (16) and sauerkraut (17). Biofilms and pellicles arise through a division of labor within microbial communities, where different members contribute distinct ECM components (18). Biofilm communities in food processing facilities were home to more than 10 bacterial species representing several phyla (19, 20). Interspecies interactions play a key role in the microbial decision to form biofilms or pellicles and in shaping biofilm morphology (14, 21).

Not all strains of *L. monocytogenes* form mono-species biofilms (6), but *L. monocytogenes* integrates into multi-species biofilms (22). The occurrence of *L. monocytogenes* in food processing facilities is positively or negatively associated with the presence of other microorganisms (23–28). Positive correlations were reported with *Pseudomonas*, *Acinetobacter*, and *Janthinobacterium* in meat processing environments and with *Enterobacter*, *Serratia*, and *Carnobacterium* in vegetable processing facilities (25, 28–30). *Herminiimonas*, *Bryobacter*, *Caulobacter*, *Sphingomonas*, and *Mycobacterium* were negatively associated with the occurrence of *L. monocytogenes* in meat processing facilities (31). However, these associations are inconsistent across commodities and facilities, and correlations do not establish causality. Experiments with reconstituted biofilms confirmed that the abundance of *L. monocytogenes* is influenced by the presence of species that facilitate survival or species that impose competitive barriers (9, 32, 33). Nevertheless, our understanding of the interaction of *L. monocytogenes* with other microorganisms in oligo-species (fewer than 10 species) or multi-species (10 or more species) biofilms remains limited, mainly owing to the complexity of an experimental design that analyzes all non-redundant combinations of strains in a microbial community with more than four or five members (9, 32). Species that facilitate or suppress *L. monocytogenes* thus remain to be identified, and very few prior studies observed the microbial decision-making on biofilms vs pellicles in diverse bacterial communities (14). It was therefore the aim of this study to determine interspecies interactions and biofilm vs pellicle formation in multi-species biofilms with or without *L. monocytogenes*. The analyses were based on a two-pronged experimental approach. First, we determined the fate of *L. monocytogenes* in six reconstituted communities that each consisted of 5–19 strains or species. Second, a 15-member community was simplified into five species, and the fate of *L. monocytogenes* in all 31 non-redundant combinations of these five strains was determined. A strain cocktail of six lineage I and II strains, each of *L. monocytogenes*, was used.

## MATERIALS AND METHODS

### Bacterial strains, culture media, and growth conditions

Six bacterial communities isolated from a meat processing facility (20) were selected to assess the presence of *L. monocytogenes* in multi-species biofilms (Table 1). Each of these communities includes all strains that were isolated from the same site of isolation at the same sampling time (20). These communities provide a reasonable representation of the core microbiota in food processing facilities (34). Plate cultures were prepared from culture stocks using Luria–Bertani agar (LB; Difco, Becton, Dickinson, Sparks, MD, USA). *Carnobacterium* spp. were cultivated on All-Purpose Tween agar (APT; Difco, Becton, Dickinson).

**TABLE 1:** Composition of six bacterial communities selected for multi-species biofilm formation with the presence of *Listeria monocytogenes*

Isolation site^*a*^	Bacterial strains^*b*^
Side cutting board (SCB)	*Serratia proteamaculans* FUA11786, *Pseudomonas gessardii* FUA11787, *Brochothrix thermosphacta* FUA30016, *Pseudomonas fragi* FUA11789, *Pseudomonas weihenstephanensis* FUA11790, *Pseudomonas helmanticensis* FUA11792, *Pseudomonas paracarnis* FUA11796, *Pseudomonas jessenii* FUA11802, *Pseudomonas lactis* FUA11804
D-drain in cooler (DDC)	*Psychrobacter* spp.* FUA11109, *Microbacterium* sp002979655* FUA2279, *Carnobacterium maltaromaticum* FUA3957, *Pseudomonas iridis* FUA11110, *Pseudomonas paracarnis* FUA11111, *Janthinobacterium* sp900112025* FUA11112, *Pseudomonas fragi* FUA11115, *Pseudomonas paraversuta* FUA11116, *Pseudomonas shahriarae* FUA11117, *Pseudomonas hygromyciniae* (sp012985665)* FUA11118, *Pseudomonas kielensis* FUA11119, *Pseudomonas* sp010095445* FUA11120, *Pseudomonas* spp.* FUA11121, *Pseudomonas* sp019345465* FUA11124, *Pseudomonas deceptionensis* FUA11126, *Pseudomonas bubulae* FUA11131, *Sphingobacterium* sp000938735* FUA11132, *Pseudomonas* sp008369295* FUA11133, *Psychrobacter cryohalolentis* FUA11134
D-little hole (DLH)	*Serratia liquefaciens* FUA11083, *Carnobacterium maltaromaticum* FUA3955, *Pseudomonas prosekii* FUA11084, *Pseudomonas iridis* FUA11087, *Pseudomonas* sp019345465* FUA11088, *Pseudomonas shahriarae* FUA11090, *Pseudomonas gessardii* FUA11091, *Pseudomonas weihenstephanensis* FUA11092, *Janthinobacterium tructae* FUA11095, *Pedobacter antarcticus* FUA11096, *Pseudomonas kielensis* FUA11097, *Pseudomonas brenneri* FUA11100, *Pseudomonas* sp008369295* FUA11102, *Janthinobacterium* sp009208735* FUA11106, *Flavobacterium* spp.* FUA11107
Drain in bagging station (DB)	*Pseudomonas rhizosphaerae* FUA11844, *Brochothrix thermosphacta* FUA30019, *Acinetobacter harbinensis* FUA11845, *Acinetobacter bohemicus* FUA11846, *Pseudomonas bubulae* FUA11848, *Serratia proteamaculans* FUA11849, *Serratia proteamaculans* FUA11850, *Pseudomonas* sp019345465* FUA11852
Surface for a break table (D-ES1)	*Pseudomonas shahriarae* FUA11001, *Pseudomonas paracarnis* FUA11002, *Pseudomonas* sp900187645* FUA11003, *Pseudomonas mohnii* FUA11005, *Pseudomonas* spp.* FUA11006, Pseudomonas iridis* FUA11007, *Aeromonas salmonicida* FUA11008, *Acinetobacter* spp.* FUA11009, *Shewanella oncorhynchi* FUA11010, *Flavobacterium bizetiae* FUA11012, *Flavobacterium* spp.* FUA11013, *Pseudomonas lundensis* FUA11015, *Pseudomonas monsensis* FUA11016
Pork picnic sample after 3 months of storage (P3M)	*Carnobacterium maltaromaticum* FUA3987, *Rahnella rivi* FUA11639, *Rahnella inusitata* FUA11640, *Serratia proteamaculans* FUA11642, *Latilactobacillus curvatus* FUA30000

^*a*^ All strains were isolated from the same site within a meat processing facility (20).

^*b*^ Strains were identified on the basis of a digital DNA–DNA hybridization of more than 70% and an average nucleotide identity (ANI) of more than 95%–96% to a validly published type strain. Strains that could not be matched to a validly published species are marked with an asterisk (*). Strains that matched a genome deposited in the Genome Taxonomy Database (GTDB) with >95% ANI are designated with a genome identifier assigned by the GTDB. If neither the validly published type strains nor the GTDB included a strain with ANI >95%, the strains are designated as spp. and FUA strain number.

For the analysis of lineage-specific traits, six strains each from lineages I and II of *L. monocytogenes* were selected (Table S1). The strains were selected based on their genomic diversity (6). Each strain was streaked on tryptic soy agar (Difco) and grown at 37°C for 48 h.

### Formation of multi-species biofilms

Biofilms were formed as described (12), with modifications to the bacterial suspension and incubation temperature. Overnight cultures of each of the 12 strains of *L. monocytogenes* were prepared individually in 5 mL of tryptic soy broth and incubated aerobically at 37°C with agitation at 200 rpm for 24 h. On the day of biofilm formation, a cocktail of the 12 strains of *L. monocytogenes* was prepared with an equal population of each strain. The optical density of the cultures at 620 nm was measured, and the cultures were diluted with LB without NaCl (LBNS) to a cell count of approximately 10⁴ CFU/mL. Overnight cultures of the six bacterial communities were prepared by incubating each strain in 5 mL of LBNS broth at 25°C aerobically for 48 h with shaking at 200 rpm, except for *Brochothrix*, *Carnobacterium*, and *Latilactobacillus* spp. *Brochothrix* spp. were cultured in 5 mL of APT broth and incubated aerobically; *Carnobacterium* spp. and *Latilactobacillus* spp. were incubated in 10 mL of APT broth and MRS6 broth, respectively, at 25°C for 2 days without agitation. To initiate biofilm formation, the strains of each bacterial community were combined in approximately equal optical densities of each strain. Aliquots of the bacterial community mixture (10 µL) and of the *L. monocytogenes* cocktail (10 µL) were added to 2 mL of LBNS broth. This suspension was then transferred into a 24-well plate (Corning, Glendale, AZ, USA), each containing a stainless steel (SS) coupon (grade 304, no. 4 finish, 12 mm diameter; Stanfos, Edmonton, AB, Canada) at the bottom, and the plates were incubated at 10°C for 7 days.

### Cell enumeration and biomass quantification in multi-species biofilms

After 7 days of incubation, biofilms were harvested for quantification of cell counts and the biofilm biomass as described (12). Pellicles were collected with sterile tweezers before collecting the biofilms on SS coupons. Pellicles and each coupon were rinsed three times with sterile distilled water to remove loosely attached cells, then pellicles and coupons were transferred into 2 mL of 0.1% peptone water containing 1.64 g of sterile glass beads (⌀ = 0.5 mm) and vortexed at maximum speed for 1 min to detach the cells. The resulting suspensions were serially diluted in 0.1% peptone water and plated on LB agar for total viable cell counts. Strains within each bacterial community were differentiated based on their colony morphology. Selective PALCAM agar with supplements and APT agar (Difco) were used for enumeration of *L. monocytogenes* and *C. maltaromaticum*, respectively. The washed SS coupons were air-dried for 25 min, and biomass was quantified in duplicate using the 1% crystal violet staining as described (12).

### Omission experiments to determine the biofilm biomass and pellicle formation with and without *L. monocytogenes*

To investigate the specific interspecies interactions driving community dynamics, the community DLH was selected. The 15-member community was simplified to a 5-member community while retaining as much of the phylogenetic diversity as possible. Biofilm formation with and without the 12-strain cocktail of *L. monocytogenes* by all 31 (2^5^-1) of non-redundant combinations of *Flavobacterium* spp. FUA 11107, *J. tructae* FUA 11095, *Pseudomonas shahriarae* FUA 11090, *Serratia liquefaciens* FUA 11083, and *C. maltaromaticum* FUA 3955 was evaluated; in addition, biofilm formation by the *L. monocytogenes* strain cocktail was quantified.

Cultures were prepared by cultivating each strain as outlined above. For each community combination, the constituent strains were mixed to achieve approximately equivalent optical densities of each strain. Biofilms were cultivated on SS coupons following the seeding protocols described above and incubated at 10°C for 7 days. After 7 days, biofilms were harvested as described (12), and differential cell counts were determined by colony morphology on non-selective LB agar, APT agar for *Carnobacterium* spp., and PALCAM agar for *L. monocytogenes*. The presence of pellicles at the air–liquid interface was recorded; pellicles were removed with sterile tweezers, washed, and dried for visual analysis. *L. monocytogenes* counts obtained on LB agar and PALCAM agar did not differ significantly (data not shown). To determine whether omission of 10 members of the DLH community alters the relative abundance of members of the community or the morphology of the biofilms, these 10 strains were sequentially added to the five-member core community with *L. monocytogenes*.

### Isolation of community DNA from biofilms

After cell enumeration, all biofilm DNA was extracted from the remaining 2 mL of biofilm suspension using the DNeasy Blood & Tissue Kit (QIAGEN, Hilden, Germany) following the manufacturer’s instructions. The purity and concentration of the DNA were measured using a NanoDrop spectrophotometer (Thermo Fisher, Waltham, MA, USA) and a Qubit fluorometer (Invitrogen, USA), respectively.

### Quantification of *L. monocytogenes* lineages I and II by quantitative PCR

Primer design for quantitative PCR (qPCR) analysis was performed with adjustments made for lineage-specific detection of *L. monocytogenes* as described (35). Genome sequences of the 12 strains of *L. monocytogenes* were downloaded from the NCBI database (6), and Roary (36) was used to select a single-copy gene that is unique to lineage I and II strains (36). Primers were designed using the PrimerQuest Tool (Integrated DNA Technologies, Coralville, IA, USA). The specificity of each primer pair was evaluated using nucleotide BLAST against the NCBI database and confirmed by PCR with template DNA isolated from 12 strains of *L. monocytogenes*.

To determine the relative abundance of lineages I and II of *L. monocytogenes* and the total *Listeria* gene copy number in all biofilms, qPCR was conducted based on established protocols (35). DNA extracted from biofilm samples was used as the template for amplification with lineage-specific primers (Table S3). qPCR assays were performed using a 7500 Fast Real-Time PCR System (Applied Biosystems, Waltham, MA, USA) with an annealing temperature of 54°C for lineage I and 58°C for lineage II. Standard curves were generated using purified PCR amplicons derived from the genomic DNA of *L. monocytogenes* FSL N3-013 (lineage I) and *L. monocytogenes* 950 008 01 (lineage II).

### Sequencing of 16S rRNA gene amplicons

To characterize the microbial composition and relative abundance within multi-species biofilms containing *L. monocytogenes*, 16S rRNA gene amplicons were sequenced using the Nanopore sequencing platform (Oxford Nanopore Technologies, Oxford, UK) following established protocols (37). Libraries were prepared with the 16S Barcoding Kit (SQK-16S114.24) using 10 ng of template DNA. Amplicons were sequenced on a MinION flow cell R10.4.1 (FLO-MIN114) for 48 h and basecalled using Dorado (v1.3.0). Sequences were analyzed on the EPI2ME platform (Oxford Nanopore Technologies) with the wf-metagenomics workflow (v2.14.1). Taxonomic classification was assigned using minimap2 (v2.28-r1209) against the ncbi_16s_18s reference database from NCBI.

### Statistical analysis

All experiments were performed in three biological repeats. Statistical analysis was performed using SigmaPlot (v12.5) and RStudio (v4.3.3). The normality of data were assessed using the Shapiro–Wilk test. Where normality assumptions were met, statistical significance was evaluated using one-way or two-way analysis of variance (ANOVA) with Tukey’s HSD post hoc analysis. For qPCR results, a two-way ANOVA with Tukey’s HSD post hoc analysis was employed to determine the effects of lineage, bacterial community, and their interaction. Post hoc comparisons were conducted using estimated marginal means with a Sidak adjustment to control for multiple comparisons (*P* < 0.05). If normality assumptions were not satisfied, a Kruskal–Wallis one-way analysis of variance on ranks was performed. For box plot correlation analyses, statistical significance was determined using a two-tailed unpaired *t*-test.

## RESULTS

### Impact of the community composition on biofilm biomass and pellicle formation

To determine the interspecies interactions of microorganisms in biofilm formation and pellicle formation, five species from the DLH community that were isolated from the same site in a pork processing facility (20) were used. The combinatorial experimental design analyzed all 31 non-redundant combinations of one to five species. All bacterial combinations formed biofilms with biomass ranging from 0.3 to 2.5 absorbance units (AU_570 nm_) and viable cell counts ranging from 6.7 to 8.6 log CFU/cm^2^ (Fig. S1). *Janthinobacterium tructae* with *Pseudomonas shahriarae* and *Carnobacterium maltaromaticum* (JPC) and the same combination with *Flavobacterium* spp. FUA11107 (FJPC) produced a higher biofilm biomass than the other groups (Fig. S1). These results indicate that community composition was the key factor influencing biofilm biomass.

**Fig 1:**
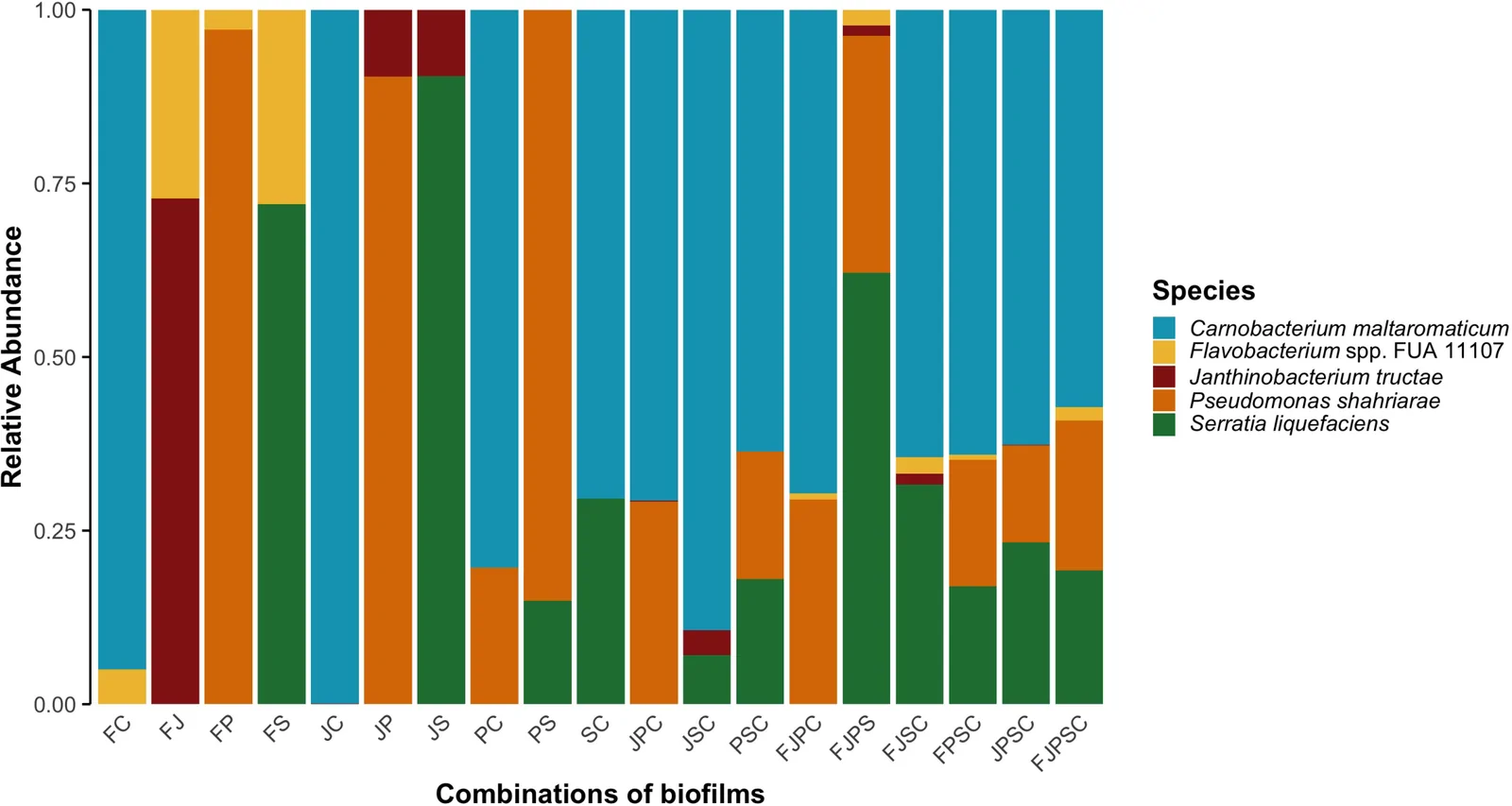
Relative abundance of the viable cell counts of five core species in different biofilm combinations. Biofilms were harvested on stainless steel coupons at 10°C for 7 days. The bar plot shows the mean relative abundance of the five strains across different combinations, including mono-, dual-, tri-, tetra-, and penta-species (FJPCS) biofilms. The relative abundance for each species within a biofilm combination was determined by differential cell counts in the average of three independent biological repeats (*n* = 3). The *x*-axis labels indicate the species present in each combination, i.e., C (*C. maltaromaticum*), S (*S. liquefaciens*), F (*Flavobacterium* spp. FUA 11107), J (*J. tructae*), and P (*P. shahriarae*), with different colors in the bar chart assigned to each species. Data are shown as means of triplicate biological repeats.

In mixed-species biofilms, *C. maltaromaticum* was highly abundant in all bacterial combinations where it was present (Fig. 1). In contrast, *Flavobacterium* spp. FUA11107 and *J. tructae* were less abundant in communities with more than two strains. In the two-species FJ combination, *J. tructae* was more abundant than *Flavobacterium* spp. FUA11107. *Serratia liquefaciens* was the most abundant species in most communities containing *Pseudomonas shahriarae*, *J. tructae*, and/or *Flavobacterium* spp. FUA11107. Overall, *P. shahriarae* was highly abundant in two- to four-species combinations where it was present; its abundance decreased when *C. maltaromaticum* was present in the community (Fig. 1).

**Fig 2:**
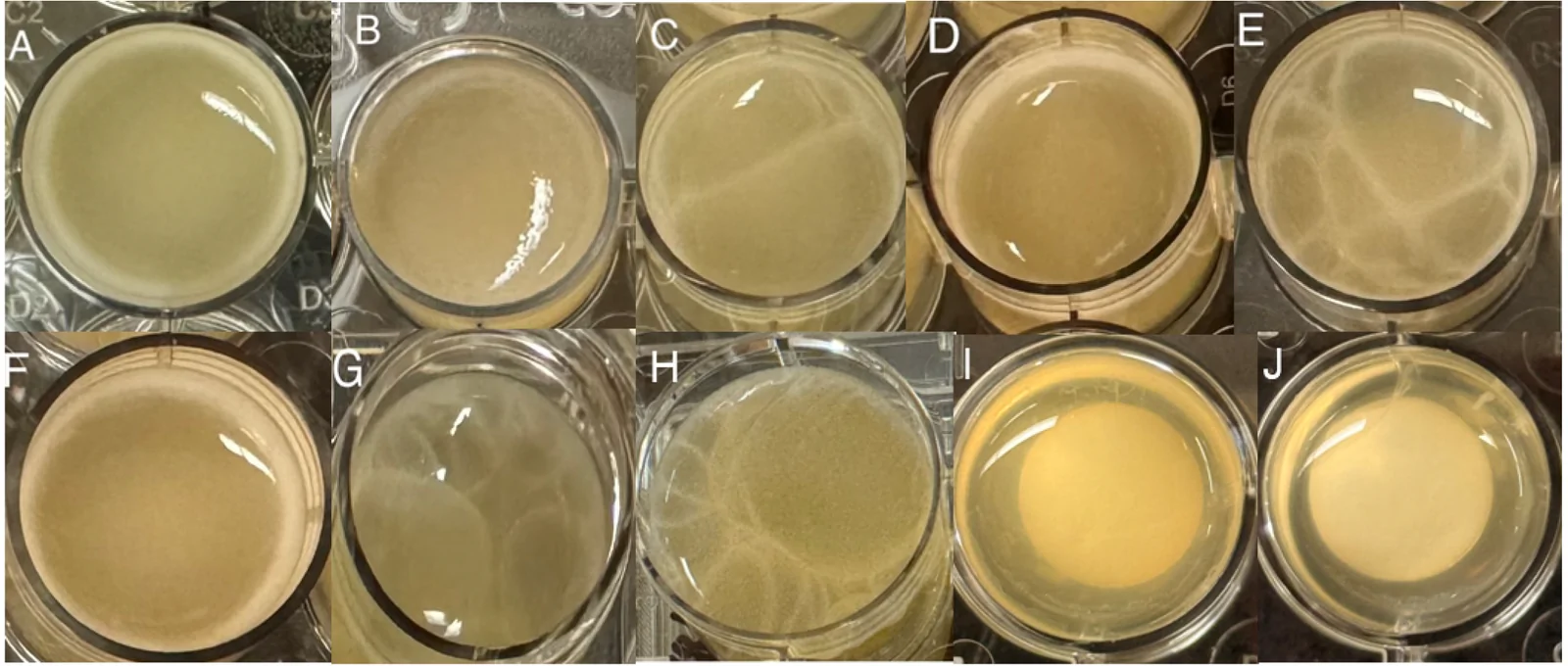
Visual assessment of pellicle formation and properties in different biofilm combinations containing *Janthinobacterium tructae*. Shown are images of biofilm and/or pellicle formation at the air–liquid interface. Biofilms were grown with the following species combinations: (**A**) J (*J. tructae*, mono-species), (**B**) FJ (*Flavobacterium* spp. FUA 111,007 + *J. tructae*), (**C**) JS (*J. tructae + S. liquefaciens*), (**D**) JC (*J. tructae + C. maltaromaticum*), (**E**) FJS, (**F**) FJC, (**G**) JSC, (**H**) FJSC, (**I**) JP (*J. tructae + P. shahriarae*), and (**J**) JPC. Images are representative of triplicate biological repeats.

The presence of *J. tructae* and *P. shahriarae* influenced decisions on pellicle vs biofilm formation. The J, FJ, JS, JC, FJS, FJC, JSC, and FJSC combinations formed pellicles (Fig. S1; Fig. 2). When *S. liquefaciens* was present with *J. tructae*, pellicles with bubbles were formed (Fig. 2), whereas the presence of *C. maltaromaticum* with *J. tructae* formed thick or thin pellicles (Fig. 2). When *P. shahriarae* was introduced into communities containing *J. tructae* (JP, JPC, FJPC, and FJPSC), biofilms rather than pellicles were formed. The JPC and FJPC combinations without *S. liquefaciens* formed thicker bottom biofilms when compared with other combinations (Fig. S1; Fig. 2). The findings further demonstrated that the composition of the bacterial community also shifts the preference for pellicles or biofilms.

### Integration of *L. monocytogenes* in six multi-species biofilms

**Fig 3:**
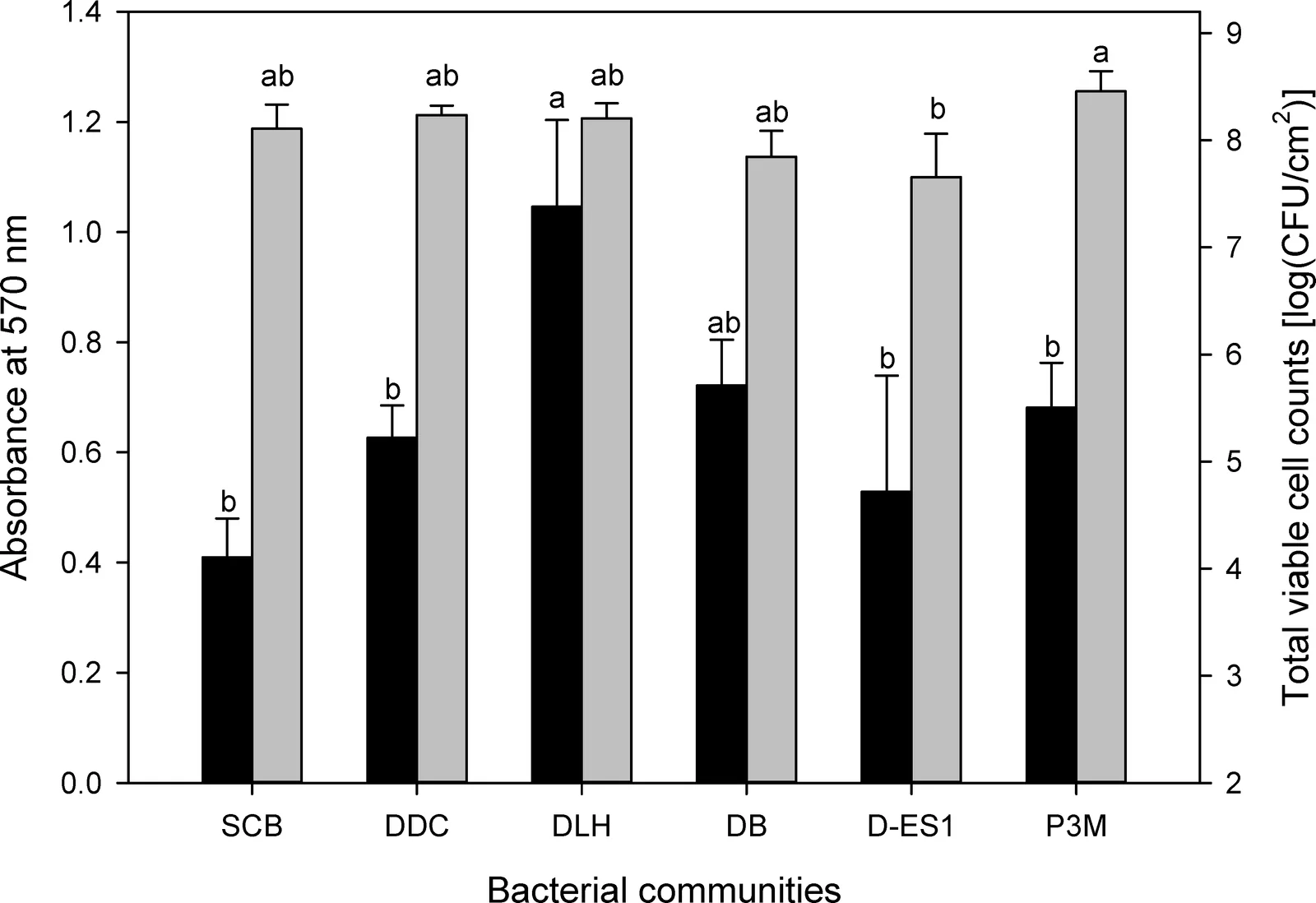
Biomass (black) and total viable cell count (light gray) of six biofilm communities with an addition of 12 strains of *L. monocytogenes*. Biofilms were incubated on stainless steel coupons at 10°C for 7 days. Biomass was quantified using 1% crystal violet staining and measured by absorbance at 570 nm. Data represent the mean ± standard deviation from triplicate biological repeats. Bars not sharing the same superscripts differ (*P* < 0.05).

To determine integration of *L. monocytogenes*, a 12-strain cocktail with strains of lineages I and II was added to six multi-species biofilms that were reconstituted with all isolates from the same site (20). Lineages III and IV were not considered as these rarely, if ever, cause foodborne disease (38). Biofilms were characterized by quantification of the biomass and differential viable cell counts (Fig. 3). Biomass and total viable cell count differ significantly among the biofilm communities (*P* < 0.001). The biomass ranged from 0.4 to 1, with community SCB exhibiting the lowest biomass and community DLH showing the highest. The total viable cell counts ranged from 7.7 to 8.5 log CFU/cm^2^ (Fig. 3).

**Fig 4:**
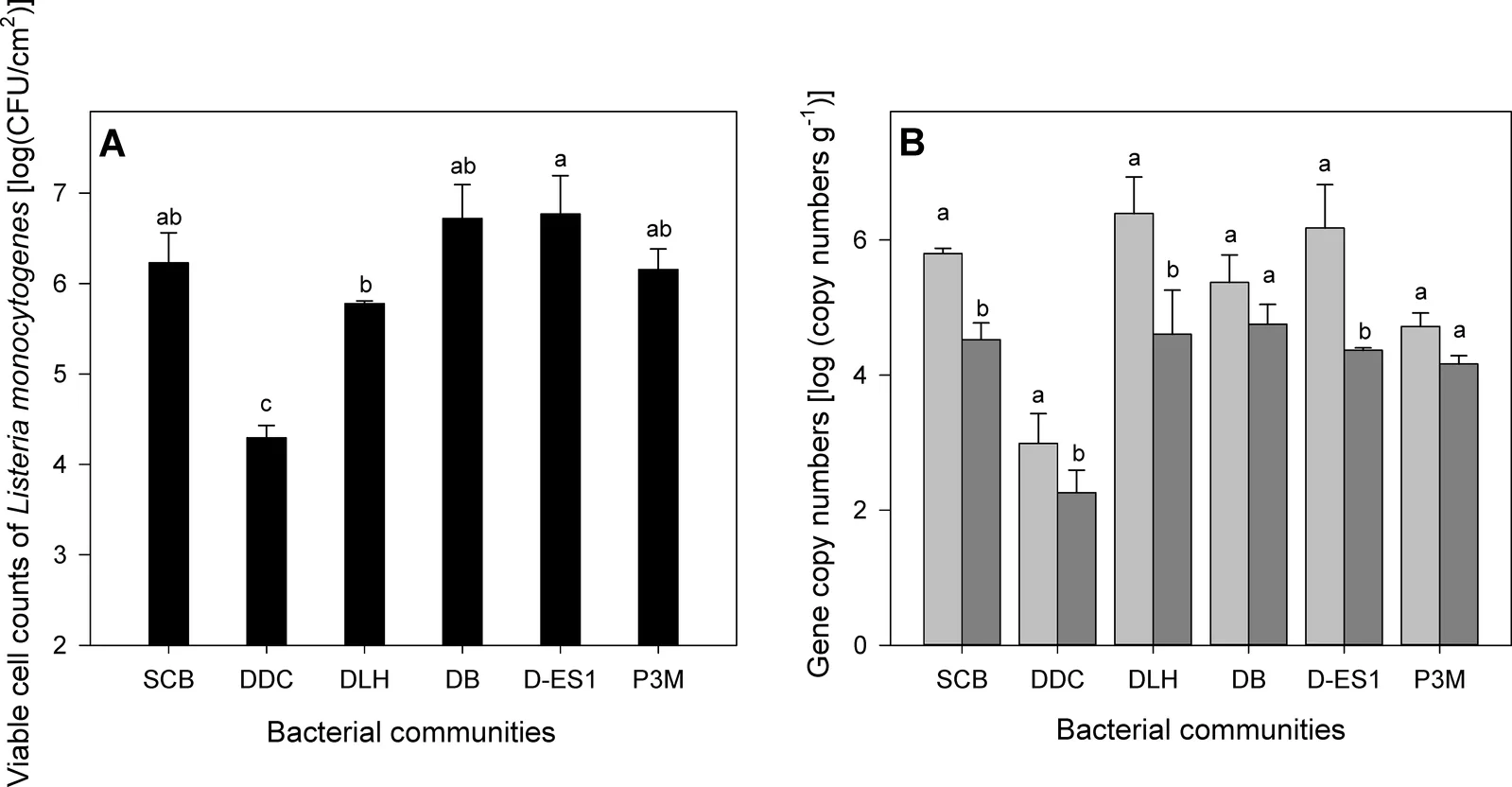
Viable cell counts and lineage-specific quantification of 12 strains of *L. monocytogenes* in lineage I (light gray) and lineage II (dark gray) across six multi-species biofilms. Biofilms were incubated on stainless steel at 10°C for 7 days. Cell counts (**A**) of *L. monocytogenes* were enumerated on PALCAM agar with a detection limit of 2 log CFU/cm^2^. Biofilm DNA was extracted using the DNeasy Blood & Tissue Kit, and lineage-specific quantification (**B**) was performed via qPCR using lineage-specific primers. The qPCR detection limit was 1.6 log copy number g⁻¹. Data are shown as means ± standard deviation of three biological repeats. Bars that do not share the same superscripts differ (*P*  <  0.05).

To assess the presence of *L. monocytogenes* in multi-species biofilms, viable cell counts were enumerated on PALCAM agar (Fig. 4). The viable cell counts of *L. monocytogenes* differed among the six biofilm communities (*P* < 0.001), ranging from 4.3 to 6.8 log CFU/cm^2^. The lowest cell counts were observed in community DDC, while the highest counts were obtained in community D-ES1 (Fig. 4A). Integration of *L. monocytogenes* into multi-species biofilms thus depends on the composition of bacterial communities.

To evaluate the lineage-specific integration of *L. monocytogenes* within multi-species biofilms, biofilm DNA samples were analyzed by qPCR targeting lineage-specific gene sequences (Fig. 4B). The composition of the bacterial community (*P* < 0.001), the lineage (*P* < 0.001), and their interactions (*P* < 0.05) influenced the abundance of *L. monocytogenes*. This interaction suggests that the presence of lineage I vs lineage II within the multi-species biofilms is contingent upon the specific composition of the bacterial community. In the majority of the six test communities, the gene copy numbers of lineage I were higher (*P* < 0.05) than the gene copy numbers of lineage II. The highest gene copies were recorded for lineage I in community DLH (6.39 log gene copies/g), 1.8 log units higher than those of lineage II in the same environment. Conversely, the lowest gene copy numbers for both lineages were observed in community DDC, 2.99 and 2.26 log gene copies/g for lineages I and II, respectively, which aligned with the lowest total viable cell counts of *L. monocytogenes*.

**Fig 5:**
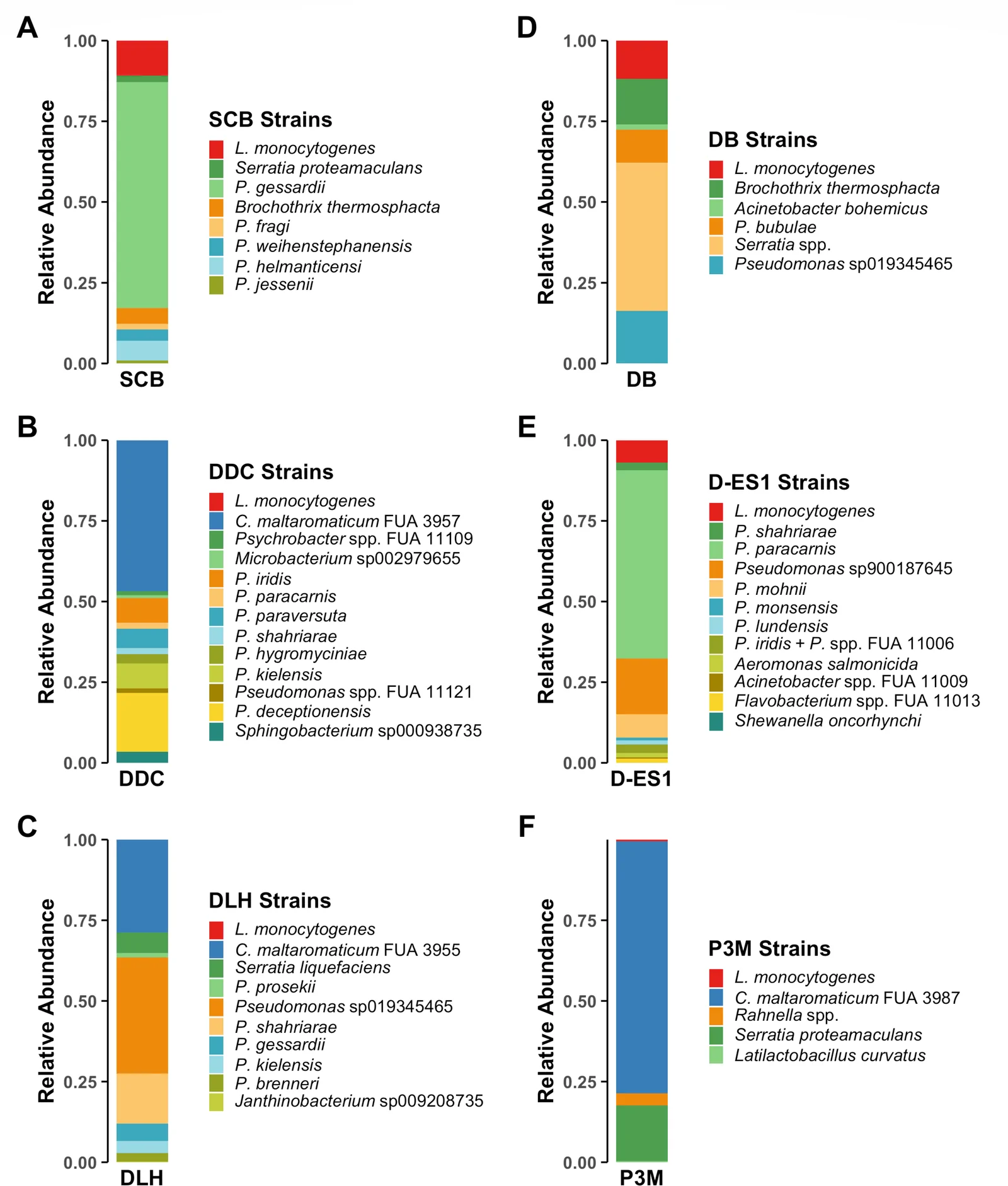
Relative abundance of bacterial strains in six biofilm communities. Biofilms were harvested on stainless steel coupons at 10°C for 7 days. Selective media were used for differential cell enumeration, including PALCAM agar for *L. monocytogenes*, APT agar for *C. maltaromaticum*, and LB agar for total viable cell counts. Stacked bar charts represent the relative abundance of strains within each biofilm community: SCB (**A**), DDC (**B**), DLH (**C**), DB (**D**), D-ES1 (**E**), and P3M (**F**). Strains are color-coded as indicated in the accompanying legend. *L. monocytogenes* was color-coded as red, and *C. maltaromaticum* was color-coded in navy blue across all bacterial communities. Data are shown as means of triplicate biological repeats.

Differential cell counts on selective and non-selective media determined the composition of the six biofilm communities. Colony morphology distinguished individual strains (Fig. 5) (37). Sequencing of 16S rRNA gene amplicons (Fig. S2) confirmed the culture-based analyses. *Pseudomonas* spp. were highly abundant in all communities, indicating their ecological fitness in diverse biofilm communities. *C. maltaromaticum* was present in communities SCB, D-ES1, and P3M. In those communities, it was among the most abundant members (Fig. 5). Its presence was consistently associated with a reduced relative abundance of *L. monocytogenes* when compared to communities without *C. maltaromaticum*.

Pellicle formation was observed in only one of six communities, community DDC (Fig. S3). The composition of the pellicle community differed from the community composition found in the biofilm. Different strains of *Pseudomonas* were dominant or present in pellicles and biofilms (Fig. 5; Fig. S3). This spatial heterogeneity suggests that while *Pseudomonas* spp. excel at colonizing the submerged solid–liquid interface, *Pseudomonas* spp. in the DDC community contribute to pellicle formation.

### Impact of increasing community complexity on pellicle formation

Reconstituted biofilms derived from the simplified five-member DLH community were assembled into all 31 non-redundant combinations and the *L. monocytogenes* strain cocktail, generating oligo-species biofilms. Only 15 of the 32 total combinations formed pellicles, and 11 were classified as thick (Table S3). Pellicle formation was consistently observed in combinations containing *J. tructae*, *P. shahriarae*, or both, suggesting that these species contribute to pellicle development. However, the final six-species biofilm community did not form a pellicle. Further analysis determined whether oligo-species biofilms in the presence of *L. monocytogenes* accurately reflected the morphology of the full 15-member multi-species community. Using the simplified five-species DLH community, we reintroduced the remaining 10 strains sequentially in pairs (Table S4). When two additional species were added to the six-species biofilm community, pellicle formation was restored. Pellicles continued to be observed until the full 15-member community was reassembled; here, no pellicle was formed. Oligo-species biofilms thus do not fully capture the complexity of the complete multi-species community.

### Impact of the community composition on cell counts of *L. monocytogenes*

**Fig 6:**
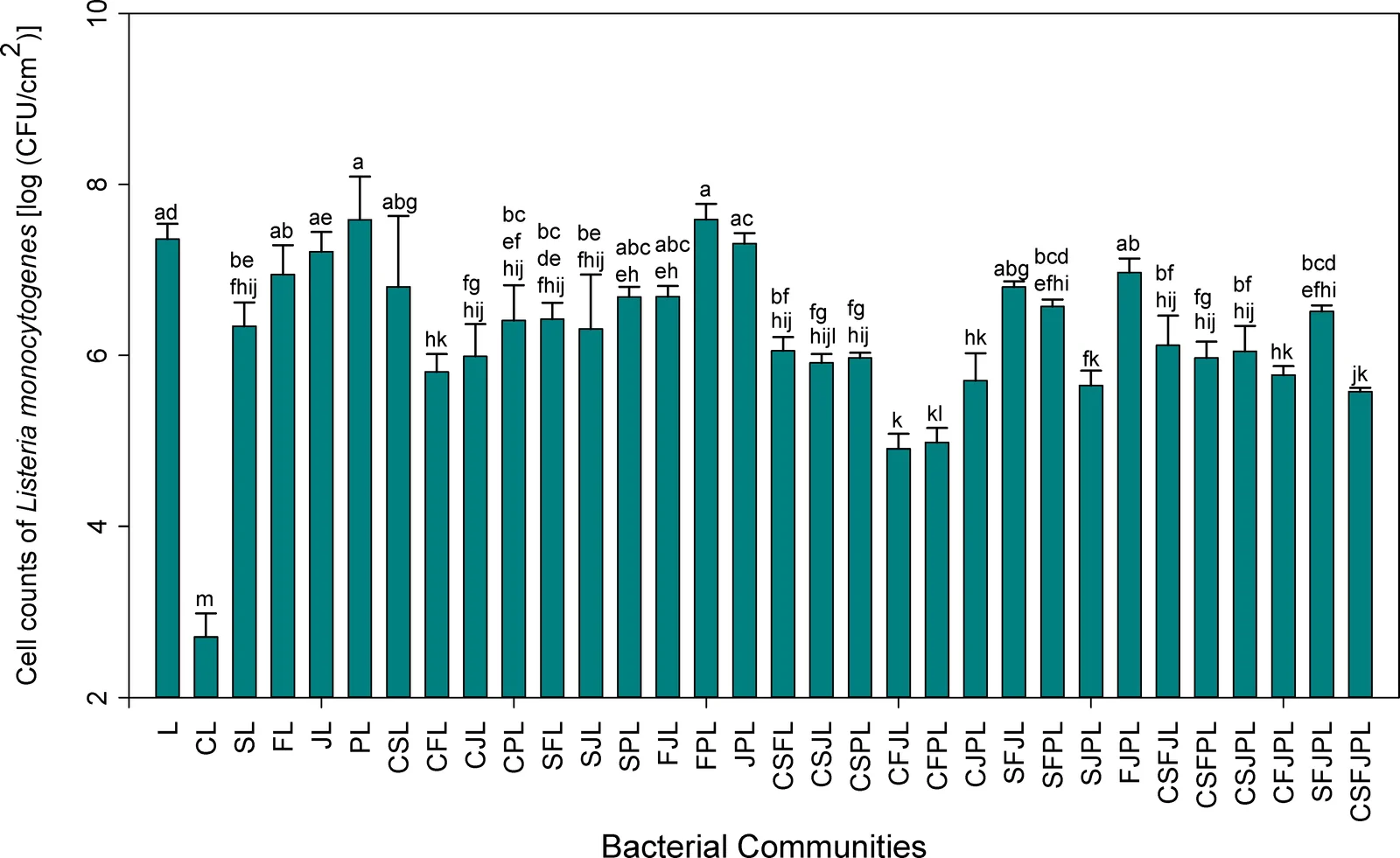
Viable cell counts of *L. monocytogenes* in biofilms formed by *L. monocytogenes* and *L. monocytogenes* with all 31 combinations of five strains. Cell counts of *L. monocytogenes* were expressed as log CFU/cm². Biofilms were formed in LBNS broth at 10°C for 7 days on stainless steel coupons. The relative abundance for each species within a biofilm combination was determined by differential cell counts in the means ± standard deviations of three biological replicates (*n* = 3). Values differ (*P* < 0.05) if the bars do not share a common superscript. The detection limit for the cell counts was 2 log CFU/cm². Each bacterial community included 12 strains of *L. monocytogenes* alone or in combination with other species: C (*C. maltaromaticum*), S (*S. liquefaciens*), F (*Flavobacterium* spp. FUA 11,107), J (*J. tructae*), and P (*P. shahriarae*).

To further examine the relationship of *C. maltaromaticum* and *L. monocytogenes* in multi-species biofilms, biofilms were grown with one to five species present in community DLH in addition to *L. monocytogenes* (Fig. 6). The 12 strains of *L. monocytogenes* formed a weak biofilm when judged by cell counts and biofilm biomass (Fig. S4). The total cell counts of the other 31 biofilms ranged from 7 to 9 log CFU/cm² (Fig. S4). *L. monocytogenes* was a minor component of the overall bacterial community in most of these 31 combinations (Fig. 4; Fig. S4 and S5). The relative abundances of genes representing lineages I and II of *L. monocytogenes* in these 31 combinations also depended on community composition. Lineage II was more abundant (*P* < 0.05) only in a few communities, e.g., FJPL, CSFJL, CSJPL, and SFJPL (Fig. S7), but the relative abundance of the two lineages did not differ (*P* > 0.05) in any of the other communities with up to five strains. As community complexity increased from 5 core members to 15 members in community DLH, the relative abundance of strains of lineage I within the biofilm increased, and gene copies representing lineage I were more abundant (*P* < 0.05) in all communities with seven or more members (Fig. S8). The cell count of *L. monocytogenes* was highest, up to 7.59 ± 0.18 log CFU/cm², in biofilms with *P. shahriarae*. In communities containing five or six strains, the cell counts of *L. monocytogenes* ranged from 5.58 ± 0.05 to 6.52 ± 0.07 log CFU/cm² (Fig. 6). The lower end of this range was observed with strain combinations that included *C. maltaromaticum*. In combinations in which it was introduced, *C. maltaromaticum* was among the dominant strains (Fig. S5). The presence of *C. maltaromaticum* reduced cell counts of *L. monocytogenes* irrespective of the presence of other species (*P* < 0.05) (Fig. 6). The inhibition was strongest in the dual-strain biofilm, where cell counts of *L. monocytogenes* were 2.71 ± 0.28 log CFU/cm² only. In the biofilm formed by *C. maltaromaticum* and *L. monocytogenes*, gene copies representing lineage I were two log cycles higher than gene copies representing lineage II, indicating differential inhibition.

**Fig 7:**
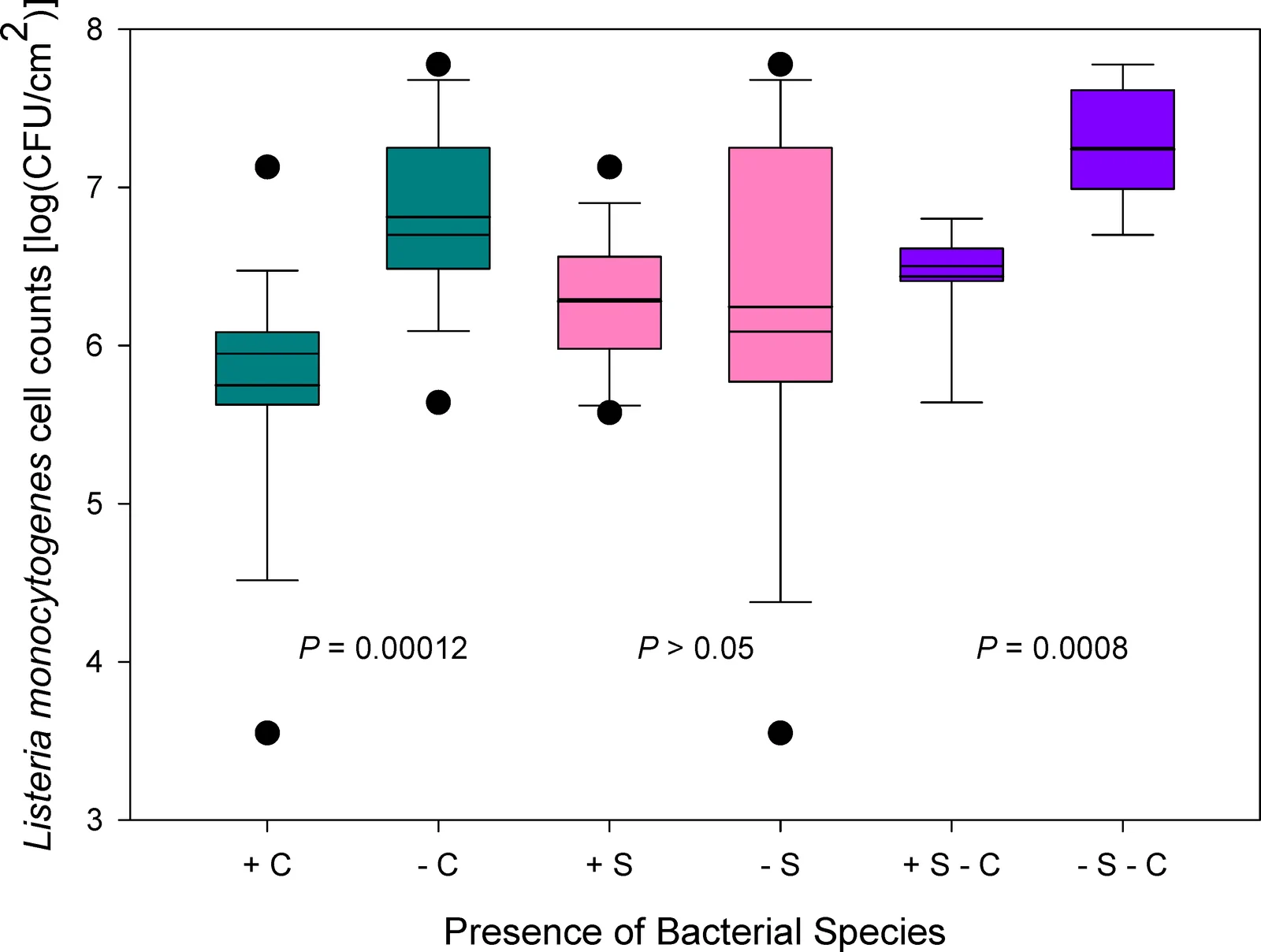
*L. monocytogenes* viable cell counts in microbial communities plotted dependent on whether or not specific other species were present. Cell counts are expressed as log CFU/cm². Biofilms were formed in LBNS broth at 10°C for 7 days on stainless steel coupons. Data represent means from three biological repeats (*n* = 3). Box plots compare *L. monocytogenes* cell counts in all communities with (+) or without (–) C (*C. maltaromaticum*) or S (*S. liquefaciens*). The cell counts of *L. monocytogenes* were also plotted for all communities without *C. maltaromaticum* with presence (+S –C) or absence (–S –C) of *S. liquefaciens*. Averages were compared with a two-tailed unpaired *t*-test. *P* values are indicated for each comparison.

To evaluate the impact of individual community members on *L. monocytogenes*, viable cell counts were compared between community combinations in which each strain was present vs absent across all 31 strain combinations (Fig. 7). The presence of *C. maltaromaticum* suppressed (*P* < 0.05) *L. monocytogenes* viable cell counts in mixed-strain biofilms compared to combinations lacking this strain. Similarly, *S. liquefaciens* reduced (*P* < 0.05) *L. monocytogenes* viable cell counts, but this effect reached significance only when excluding communities with *C. maltaromaticum*. No effect (*P* > 0.05) of *S. liquefaciens* was observed when *C. maltaromaticum* was present. The presence of *Flavobacterium* spp., *J. tructae*, and *P. shahriarae* did not influence *L. monocytogenes* viable cell counts (*P* > 0.05) (Fig. S6).

## DISCUSSION

### Experimental design and community complexity

The ecology of biofilm formation and pellicle formation differ between multi-species bacterial communities and their individual constituents. The experimental design of this study utilized a (2*^n^* − 1) combinatorial approach to evaluate all non-redundant combinations of five species for biofilm biomass, pellicle formation, and antagonism of *L. monocytogenes*. A comparable approach was previously used with biofilm communities comprising up to four members (39–41). While a community with 15 members better reflects the complexity of the microbial communities in food processing environments than communities with 3-5 members (20, 25), such a design necessitates 32,767 combinations. A reduction to a five-member community while maintaining most of the phylogenetic diversity was thus necessary to obtain a feasible experimental design. This reduction is supported by the keystone species hypothesis, which suggests that community functionality and biofilm formation are often determined by only a few dominant taxa, e.g., *Microbacterium lacticum* and *Paenibacillus amylolyticus* (39–41). Our results identified key species that prefer pellicle formation at the air–liquid interface, i.e., *J. tructae*, while other organisms, particularly *P. shahriarae*, argue successfully in favor of building biofilms. The limits of simplification were revealed when comparing the oligo-species 5-member community to the full 15-member multi-species community DLH. An increased complexity of the community altered the biofilm phenotype from pellicle formation to no pellicle formation when *Listeria* was additionally present, highlighting that higher-order interactions emerge as diversity scales.

### Pellicle formation by different microbial communities

Pellicles represent a distinct biofilm phenotype that develops at the air–liquid interface (14, 15, 42). *J. tructae* formed pellicles at the air–liquid interface rather than surface-associated biofilms. This preference for growth at the air–liquid interface is consistent with other strict aerobes, such as acetic acid bacteria, which form pellicles to maximize oxygen availability (14). In *Escherichia coli*, the transition from a planktonic state to a biofilm is well documented. In *E. coli*, pellicle formation is associated with the accumulation and spatial organization of extracellular polysaccharides, including poly-β-(1→6)-*N*-acetylglucosamine, cellulose, and colanic acid (43–45). The biofilm phenotype is determined not simply by the presence of these components but by their chemical modification and relative abundance (44, 46). The “switch” between biofilm and pellicle in *E. coli* is regulated by fine-tuning the ECM (46). For example, chemical modification of cellulose to phosphoethanolamine cellulose is essential for pellicle formation (45, 47). Minor changes in the ratio of this modified cellulose (carbohydrate) to curli (protein) also regulate this switch (46). Additionally, pellicles exhibit a greater abundance of type 1 fimbriae compared to biofilms (44, 48). The presence of other organisms influences decision-making. In dual-species biofilms, interspecies interactions mediated by quorum-sensing signals influenced the transition from pellicle to biofilms (14). Specifically, communication with a LuxS type AI-2 quorum-sensing system by *Aeromonas australiensis* convinced a pellicle-forming strain of *E. coli* to join a biofilm (14).

**Fig 8:**
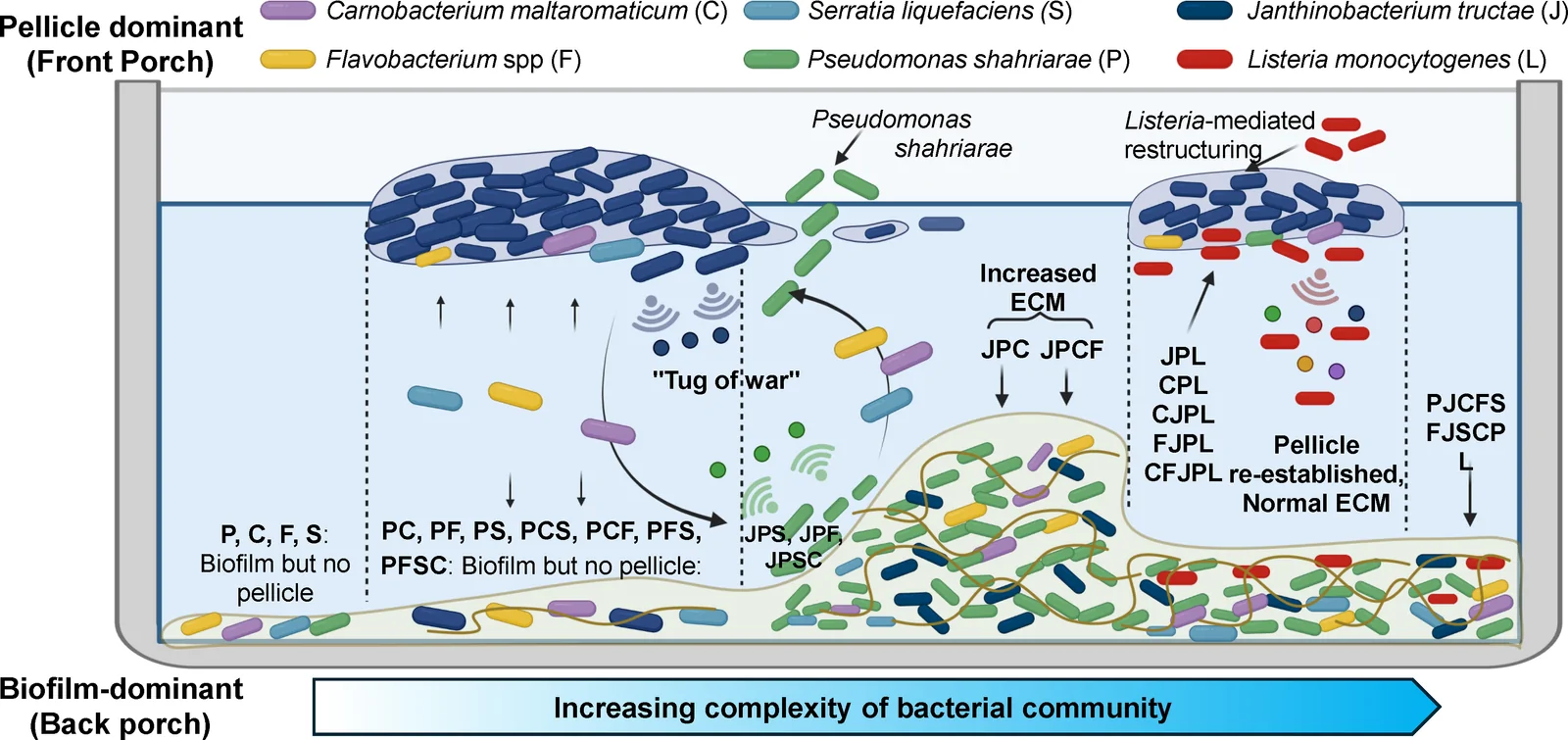
Overview of bacterial interactions that influence the decision on pellicle formation vs biofilm formation. The figure design, scientific content, and all graphical elements were created, refined with BioRender, and manually edited by the authors to obtain the final version.

Intense communication among many species with a multitude of quorum sensing systems creates a “tug-of-war” effect (Fig. 8). *P. shahriarae* formed surface-associated biofilms, while *J. tructae* preferred pellicle formation. Most other members of the community joined either *P. shahriarae* at the bottom or *J. tructae* at the top. Negotiations between *P. shahriarae* and *J. tructae* resulted in a surface-associated biofilm. When *C. maltaromaticum* joined the PJ community, communication became more complex and dynamic, resulting in a surface-associated biofilm with a substantially higher biomass but also a higher variability (Fig. S1 and S5). Likewise, a high but variable biofilm biomass was observed in the quaternary community consisting of *P. shahriarae*, *J. tructae*, *C. maltaromaticu*m*,* and *Flavobacterium* spp. (Fig. S1 and S5). This shift is likely attributable to an increased production of ECM (39, 49). As the community becomes more complex with four or more species present, a larger and more structured biofilm matrix supports distinct niches where each species occupies its own preferred micro-niche (50). The introduction of *L. monocytogenes* changed the balance of pellicle vs surface-associated biofilm again (Fig. S1 and S4). Shifts in biofilm biomass and location reflect complex communication between facultative anaerobes, such as *L. monocytogenes* and *C. maltaromaticum*, and strict aerobes, including *J. tructae* and *P. shahriarae* (20, 51, 52). These interactions may alter oxygen and nutrient availability, driving spatial reorganization of the community (53, 54). Overall, the dynamics in multi-species biofilms can be likened to a house-warming party where one resident prefers the front porch, whereas the other prefers the backyard. Each incoming guest changes whether the party is held at one site, the other, or both.

### Quorum sensing: a putative mechanism for cooperativity and antagonism

To document the complexity of communication in a community of 15 bacterial strains, their genomes were queried for quorum-sensing systems, including *N*-Acyl homoserine lactones (AI-1), which enable communication among gram-negative bacteria (55); autoinducing peptides, which enable communication among gram-positive bacteria (56); furanosyl borate diester (AI-2), which enables communication among all bacteria (57); and 3,6-dimethylpyrazin-2-one (AI-3), which enables interkingdom communication (58). Collectively, the genomes of the 15 strains encoded for more than 90 putative quorum-sensing systems, and each member of the community except *Pedobacter antarcticus* encoded for at least one (Tables S5 and S6). Strains of *Pseudomonas* encoded for multiple genes associated with AI-3 signaling through the QseB system, whereas AI-2-related signaling was detected in *Serratia* and *Carnobacterium* (Table S5). The *Carnobacterium* encoded for the highest number of quorum-sensing-related genes among gram-positive bacteria (19). This complexity of bacterial communication among diverse bacteria in multi-species biofilms all but precludes a targeted approach with k.o. mutants, that previously unraveled communication on biofilm or pellicle formation between *A. australiensis* and *E. coli* (14), or the use of membrane-based Transwell systems that determined the role of AHL-based quorum-sensing systems in triple-species biofilms (59). The expression of quorum-sensing-related genes and the identification of quorum-sensing molecules remain subject to future work that likely requires multiple “omics” approaches (19, 60, 61).

### Integration of *L. monocytogenes* in multi-species biofilms

We quantified the relative abundance of strains of lineages I and II across all biofilm conditions, but the observed patterns were inconsistent across the different biofilm communities, which suggests that the lineage-associated differences are not major determinants of the integration of *L. monocytogenes* in multi-species biofilms. Our work also extends current knowledge (25, 62) by identifying *C. maltaromaticum* and *S. liquefaciens* as biofilm residents that antagonize *L. monocytogenes*. The differential cell counts of *L. monocytogenes* in communities with *C. maltaromaticum* and *S. liquefaciens* were relatively small, about 1 and 0.5 log CFU/cm^2^ reductions, respectively. However, such modest differences in a single batch experiment may underestimate the true impact of microbial antagonism on biofilms in industrial settings, where microorganisms experience a continuous series of growth–sanitation–regrowth cycles (63–65). The final viable cell counts of *L. monocytogenes* were lower than the initial inoculum in several of the bacterial communities, therefore, even small differences or minor competitive advantages or inhibitory effects are amplified over time (66–68).

Only a few reports describe antagonistic activity of *Serratia* spp. against *L. monocytogenes* (69), but these do not relate to multi-species biofilms. Conversely, the antagonism of *C. maltaromaticum* against *L. monocytogenes* is well described (70–72). The inhibitory effects relate to both the formation of organic acids and the production of class I and class II bacteriocins (73–75). *C. maltaromaticum* is used commercially as a biopreservative to inhibit the growth of *L. monocytogenes* on ready-to-eat foods (74–76). When residing in biofilms, *Carnobacterium* spp. also expressed bacteriocin-related genes (19). In dual-species biofilm CL, gene copy numbers of strains from lineage II were two log units lower than strains from lineage I (Fig. 6; Fig. S6), indicating that the sensitivity of *L. monocytogenes* to bacteriocin-mediated inhibition is lineage dependent.

The competitive advantage of *C. maltaromaticum* over *L. monocytogenes* may be reinforced by quorum-sensing mechanisms (77, 78). The expression of quorum-sensing-related genes in *Carnobacterium* may modulate the behavior of *L. monocytogenes* through interspecific cross-talk (58, 78, 79). Many bacteriocins of lactic acid bacteria also function as quorum-sensing systems; i.e., antagonistic activity and interspecies communication are intricately linked (Table S6) (80, 81). The CS–CbnK–CbnR system of *C. maltaromaticum* LV17 controls population-density-dependent expression of carnobacteriocin and immunity genes, supporting a model in which peptide signaling and bacteriocin production act together to enhance ecological fitness and antagonistic activities (Table S7) (82, 83). High levels of AI-2 or peptides produced by *Carnobacterium* interfere with the *Listeria agr* system, the primary quorum-sensing circuit in *L. monocytogenes* (78, 84). Lactic acid bacteria produce secreted proteins with potential quorum-sensing inhibitory activity against *L. monocytogenes* (35, 36, 39–41). This molecular interference of communication systems of *L. monocytogenes* may contribute to suppressing its growth (19).

In conclusion, microbial communication in relation to biofilm vs pellicle formation and on whether to accommodate *Listeria or not* partially converge with quorum-sensing systems as a key mechanism. The analogy of biofilm formation and a housewarming party partially holds: as more guests (bacteria) arrive, the social dynamics and the locations of the party shift. This complexity is further shaped by the interplay of languages that communicate both friendly (cooperative) and confrontational (antagonistic) content. A common language coordinates broadly across the house, while private dialects that outsiders cannot understand are used as well. Results presented in this study document that the use of protective cultures may also reduce persistence of *Listeria* in food processing facilities. The novel perspective on microbial interactions in biofilms may direct future experiments. For now, it is time for a cold one, but we remain with a question: on the front porch or the back porch?

## SUPPLEMENTAL MATERIAL

Supplemental material aem.01386-26-s0001.pdf Fig. S1 to S8; Tables S1 to S7.

ASM does not own the copyrights to Supplemental Material that may be linked to, or accessed through, an article. The authors have granted ASM a non-exclusive, world-wide license to publish the Supplemental Material files. Please contact the corresponding author directly for reuse.
