# Supplementary material for: Welcome to the house-warming party: social interactions and antagonism against Listeria monocytogenes in multi-species biofilms
Source: Appl Environ Microbiol. 2026 Sep 30;92(10):e01386-26. doi: 10.1128/aem.01386-26 (PMC13689444; doi:10.1128/aem.01386-26)
Supplement: Supplementary file 1 [file aem.01386-26-s0001.pdf]

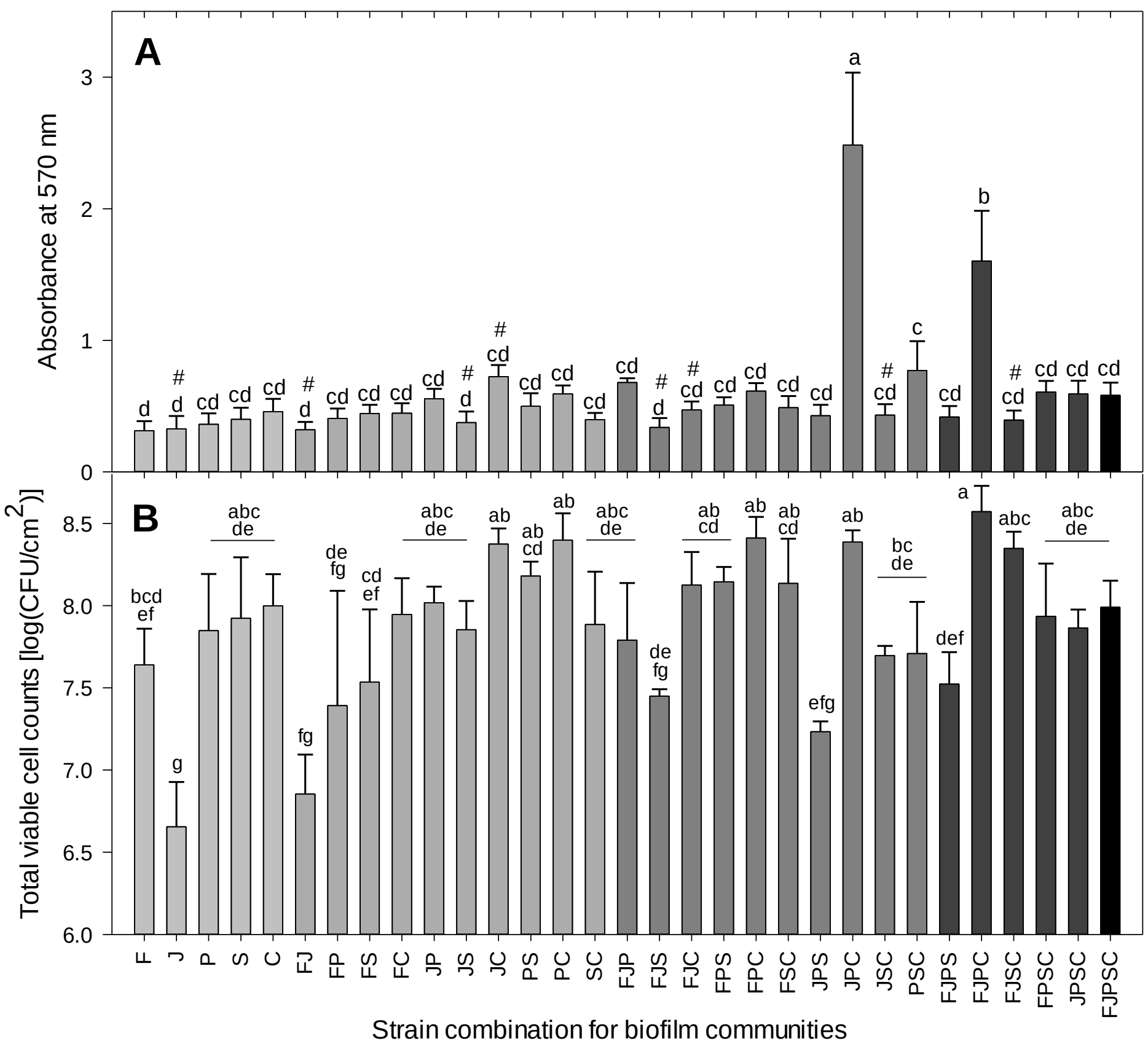

Figure S1. Biomass (**Panel A**) and total viable cell count (**Panel B**) of biofilms by different combinations of the five core bacterial species *Carnobacterium maltaromaticum*, *Flavobacterium* spp. FUA 11107, *Janthinobacterium tructae*, *Pseudomonas shahriarae*, and *Serratia liquefaciens*. Biofilms were harvested on stainless steel coupons at 10 °C for 7 d. The biomass of biofilms was quantified using crystal violet staining, and absorbance was measured at 570 nm. Viable cells were enumerated on LB agar. Bars represent means ± standard deviation of three independent experiments (n = 3). Within each panel, bars that do not share the same superscript letter differ significantly ($P < 0.05$). Communities that formed a pellicle in addition to the surface-attached biofilm are marked with #. Bar colour denotes the number of species in biofilm formation, from single-species (light grey) to penta-species communities (black).

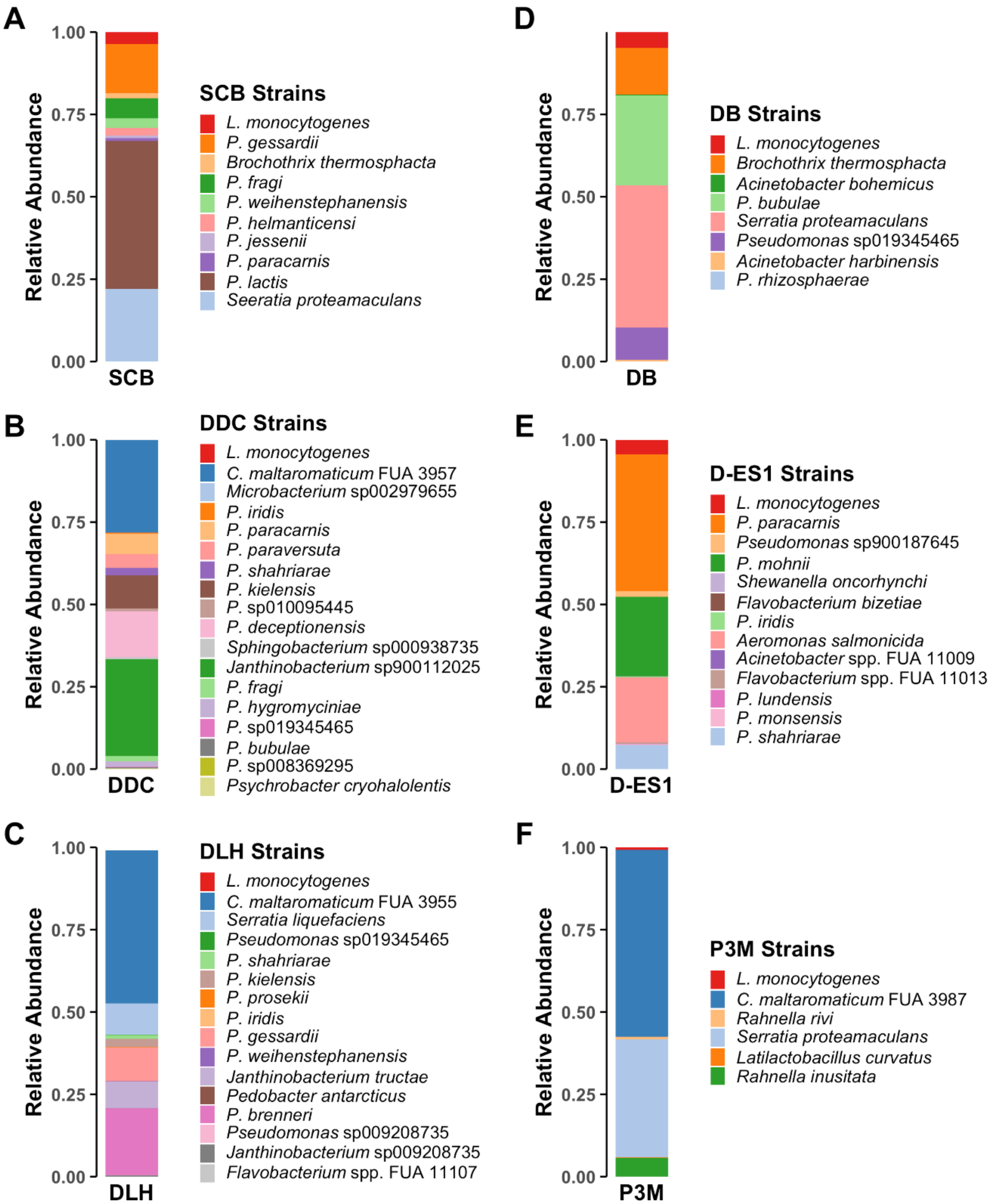

Figure S2. Relative abundance of bacterial species as determined by sequencing of 16S rRNA gene amplicons from six biofilm communities. Biofilms were harvested on stainless steel coupons at 10 °C for 7 d, and DNA samples of biofilms were extracted using Blood & Tissue Kit. 16S rRNA gene amplicon sequencing was conducted using ONT Nanopore sequencing. Stacked bar charts represent the relative abundance of strains within each biofilm community: SCB (A), DDC (B), DLH (C), DB (D), D-ES1 (E), and P3M (F). Strains are colour-coded as indicated in the accompanying legend.

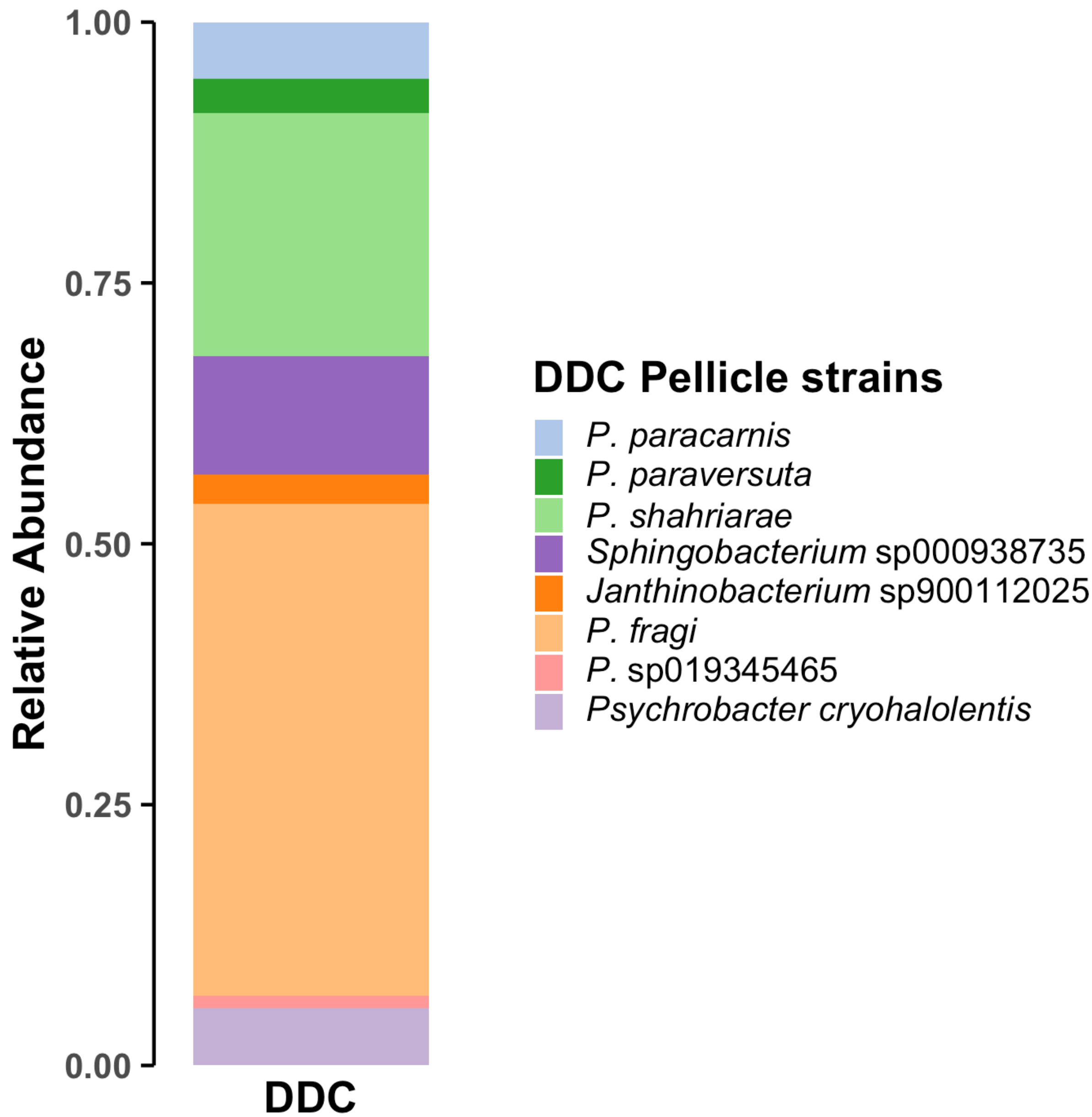

Figure S3. Relative abundance of viable cell counts of specific species in the pellicle formed by community DDC. Pellicles were harvested on the air-liquid interface grown at 10 °C for 7 d. Differential cell counts were enumerated on LB agar based on colony morphology. Stack bar charts represent the average relative abundance of strains within DDC community in three biological repeats (n=3). Strains are color-coded as indicated in the legend.

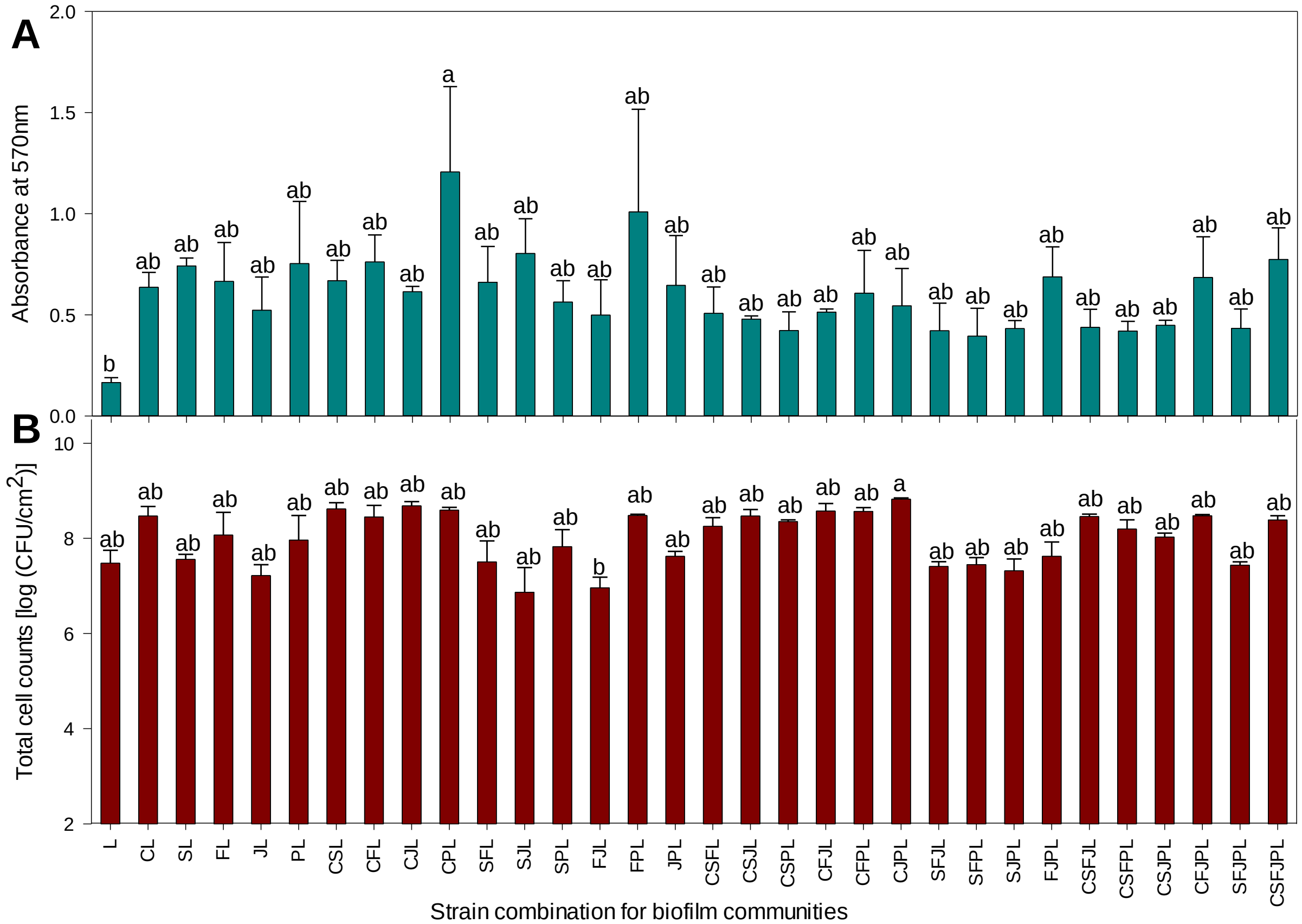

Figure S4. Biomass quantification (**Panel A**) and total cell counts (**Panel B**). Biomass was quantified by absorbance measurements at 570 nm, while total cell counts were expressed as log(CFU/cm²). Biofilms were formed in LBNS broth at 10ºC for 7 days on stainless steel coupons. Data are shown as mean ± SD from three biological replicates (n=3). Values differ significantly (P<0.05) if the bars do not share a common superscript. The detection limit for the cell counts is 2 log(CFU/cm²). Each bacterial community includes 12 strains of *L. monocytogenes* alone or in combination with other species: C (*C. maltaromaticum*), S (*S. liquefaciens*), F (*Flavobacterium* spp. FUA 11107), J (*J. tructae*), and P (*P. shahriarae*).

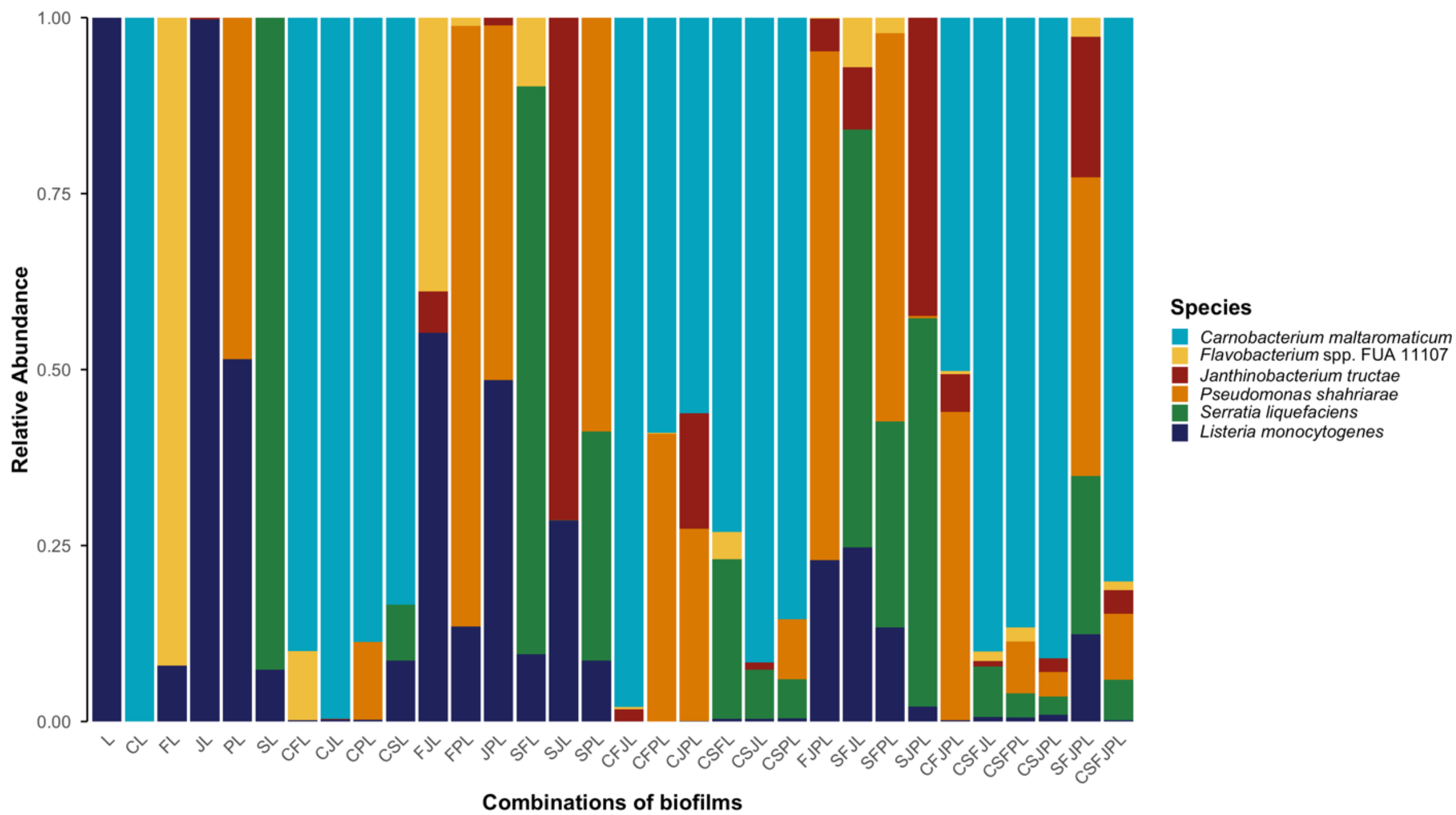

Figure S5. Relative abundance of viable cell counts of bacterial species in the different bacterial communities with *L. monocytogenes*. Biofilms were formed in LBNS broth at 10ºC for 7 days on stainless steel coupons. The relative abundance for each species within a biofilm combination was determined by differential cell counts in the average of three independent biological repeats (n = 3). Colors in the bar graph correspond to specific bacterial species as indicated in the legend on the right. Each bacterial community includes 12 strains of *L. monocytogenes* alone or in combination with other species: C (*C. maltaromaticum*), S (*S. liquefaciens*), F (*Flavobacterium* spp. FUA 11107), J (*J. tructae*), and P (*P. shahriarae*). The detection limit for *L. monocytogenes* was 2 log (CFU/cm^2); the detection limit of all other organisms was 1% of the total cell counts or about 6 log CFU/cm².

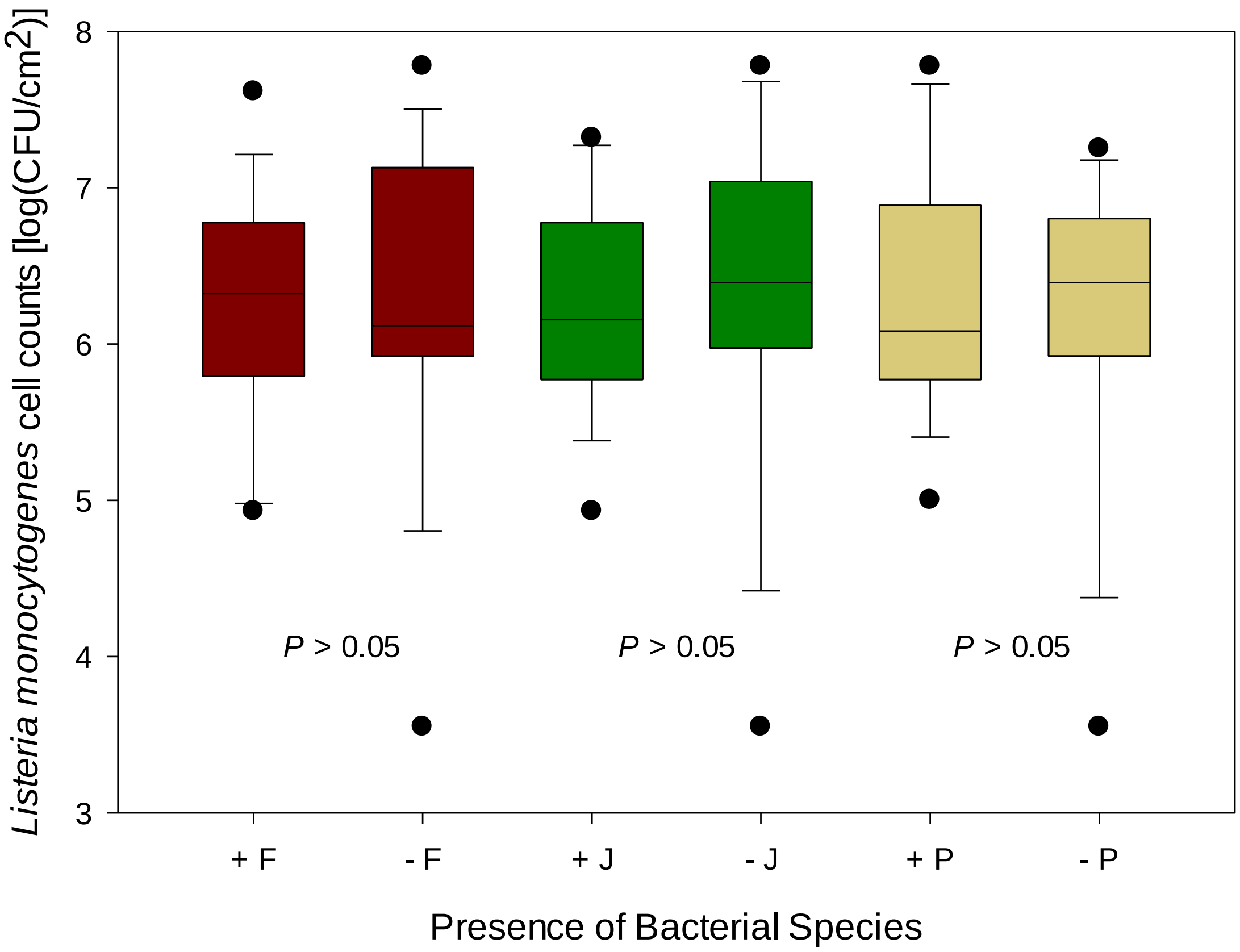

Figure S6. Variation in *L. monocytogenes* viable cell counts based on the presence or absence of other species. Cell counts are expressed as log(CFU/cm²). Biofilms were formed in LBNS broth at 10ºC for 7 days on stainless steel coupons. Data represent means from three biological replicate experiments (n=3). Box plots compare *L. monocytogenes* cell counts in the presence (+) or absence (–) of: F (*Flavobacterium* spp. FUA 11107), J (*Janthinobacterium tructae*), and P (*Pseudomonas shahriarae*). Mean values were compared with a two-tailed unpaired t-test. *P*-values are indicated for each comparison.

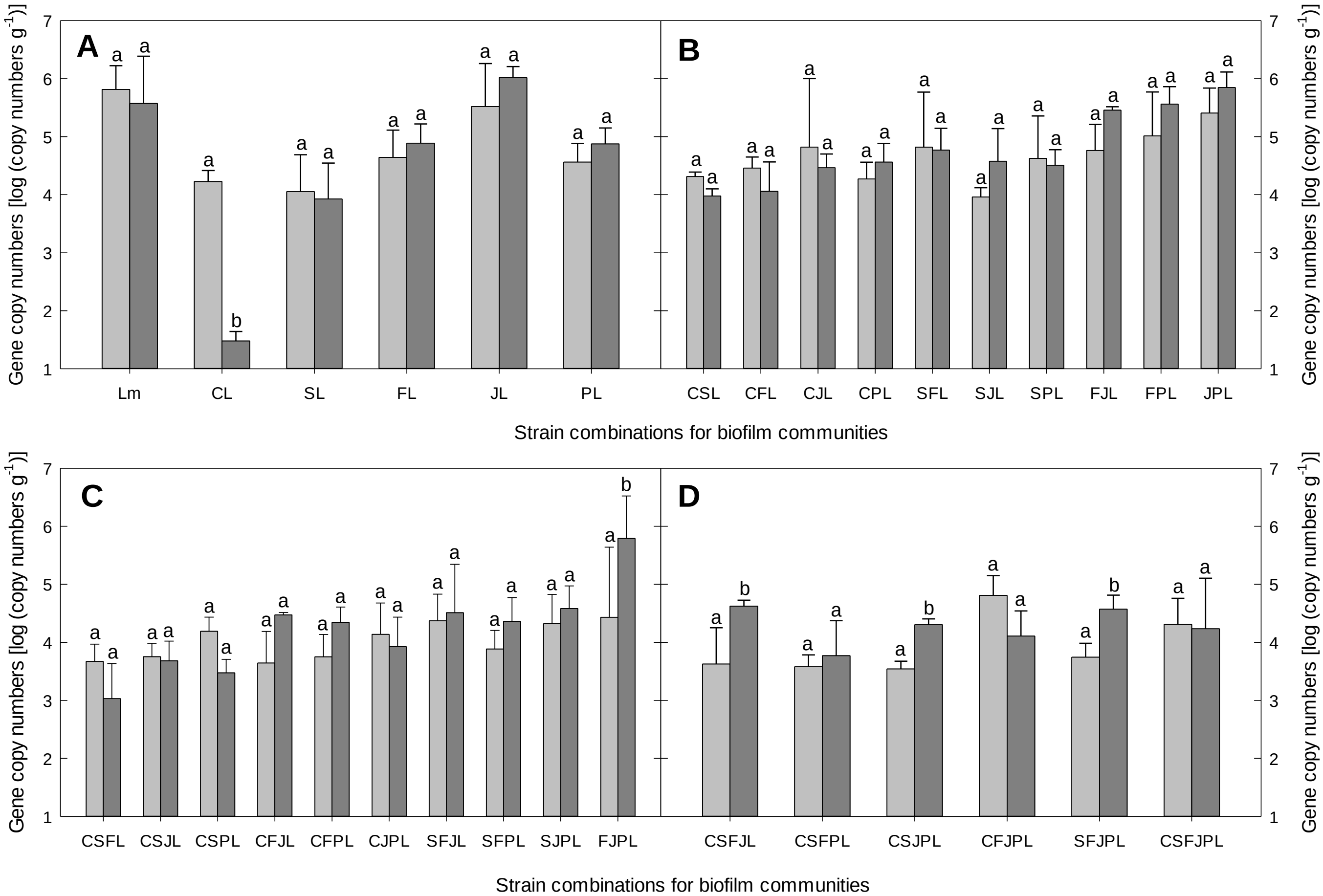

Figure S7. Lineage-specific quantification of 12 strains of *L. monocytogenes* in Lineage I **(light grey)** and Lineage II **(dark grey)** in biofilms formed by *L. monocytogenes* and *L. monocytogenes* with all 31 combinations of 5 strains. Biofilms were incubated on stainless steel at 10 for 7 d. Biofilm DNA was extracted using the DNeasy Blood & Tissue Kit and quantified via qPCR using Lineage-specific primers. (A) Mono-species combinations with *L. monocytogenes* alone. (B) Dual-species combinations with *L. monocytognes*. (C) Three-species consortia. (D) Four- and five-species combinations with *L. monocytogenes*. . Each bacterial community includes 12 strains of *L. monocytogenes* alone (L) or in combination with other species: C (*C. maltaromaticum*), S (*S. liquefaciens*), F (*Flavobacterium* spp. FUA 11107), J (*J. tructae*), and P (*P. shahriarae*). The detection limit of qPCR was 1.6 log (copy number g^{-1}). Data represent means ± SD from three independent experiments (n = 3). Bars that do not share the same superscripts differ significantly ($P < 0.05$).

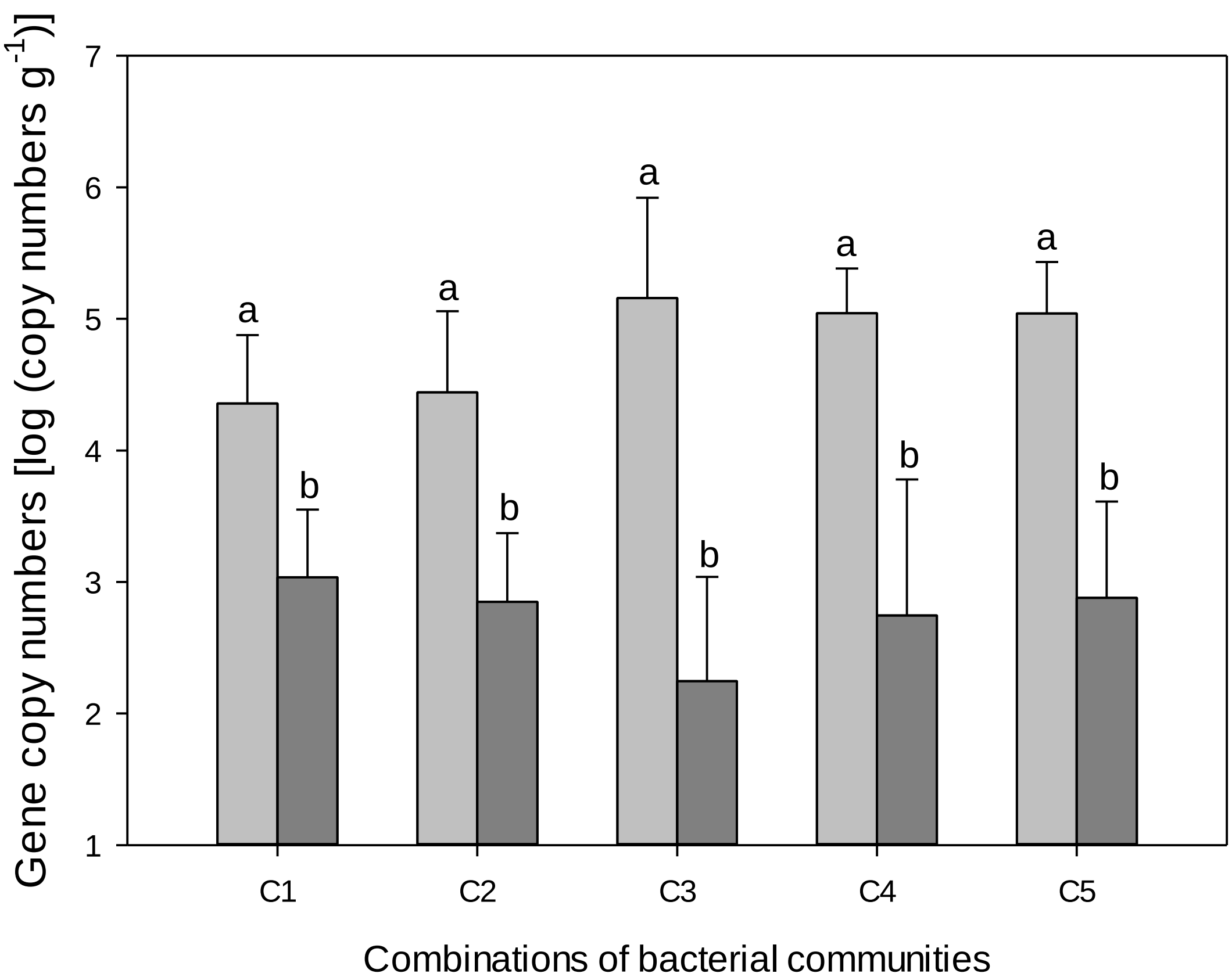

Figure S8. Lineage-specific quantification of *L. monocytogenes* across increasing bacterial community complexities. Abundance of *L. monocytogenes* Lineage I **(light grey)** and Lineage II **(dark grey)** within expanding community combinations (C1–C5). The core community (C1: CSFJPL) was sequentially supplemented with two additional strain to reach a maximum complexity of 15 members in community DLH (C5). Biofilms were established on stainless steel at 10°C for 7 days. Biofilm DNA was extracted using DNeasy Blood & Tissue Kit and quantified via qPCR using lineage-specific primers. The detection limit of qPCR was 1.6 log (copy number g^{-1}). Community compositions were as follows: C1: CSFJPL core; C2: C1 + *P. prosekii* and *P. iridis*; C3: C2 + *Pseudomonas* sp019345465 and *P. gessardii*; C4: C3 + *P. weihenstephanensis* and *P. kielensis*; C5: C4 + *P. brenneri*, *Janthinobacterium* sp009208735, *P.* sp008369295, and *Pedobacter antarcticus*. Data represent means ± SD from three independent experiments (n = 3). Bars that do not share the same superscript letters differ significantly ($P < 0.05$).

Table S1. 12 strains of *Listeria monocytogenes* used in this study [3].
* MAPAQ: Ministère de l’Agriculture, des Pêcheries et de l’Alimentation du Québec, Canada.

Strain designation	Lineage	Source	Serogroup	CC	NCBI Biosample accession number	Origin or Reference
HPB 394	I	Cheese	IIb	CC3	JBALBJ000000000	Health Canada
HPB 2130	I	Chocolate milk	IIb	CC3	JBALBH000000000	Health Canada
FSL J1-177	I	Human (sporadic)	IIb	CC288	SAMN53284150	[1]
FSL N3-013	I	Pate	IVb	CC2	SAMN53284151	[1]
FSL N1-227	I	Food (epidemic)	IVb	CC6	SAMN53284153	[1]
HPB 1883	I	Soft unpasteurized cheese	IVb	CC1	JBALBD000000000	Health Canada
HPB 2921	II	Environmental swab	IIc	CC9	JBALAC000000000	Health Canada
FSL R2-499	II	Sliced turkey	IIa	CC11	SAMN53284152	[1]
FS 12	II	Fermented sausage	IIa	CC8	JBALAI000000000	[2]
950 008 01	II	Raw milk	IIa	CC89	JBALAR000000000	MAPAQ*
454 046 01	II	Raw cheese milk	IIa	CC11	JBALAM000000000	MAPAQ
934 294 02	II	Cheese	IIa	-	JBAMDL000000000	MAPAQ

Table S2. Lineage-specific primers and species-specific primers used in this study for qPCR quantification of *Listeria monocytogenes* Lineage I and Lineage II strains, and total *L. monocytogenes* in all biofilm communities.
biofilms.

**Targeted lineage**	**Gene**	**Primer**	**Sequence (5' - 3')**	**Amplicon length (bp)**
L. monocytogenes	*hlyA*	LMA*<br>LMB*	CGGAGGTTCCGCAAAAGATG<br>CCTCCAGAGTGATCGATGTT	234
Lineage I	*llsA*	llsA-F<br>llsA-R	TGC TGT AGG CTC TGA AGC AAT<br>CAT TTT GGT TGC AGC AGA TGA T	106
Lineage II	*bglF_2*	bglF_2-F<br>bglF_2-R	TGG CAA TAC CGT TGG AGA TG<br>CTA TCA GCA GCC GCG TAT AAA	270

*: Primers marked with an asterisk were taken from [4].

Table S3. Summary of pellicle formation by different bacterial communities, including their presence and thickness (thick or thin).

Combination	Thickness	Combination	Thickness	Combination	Thickness
L	-	CSL	-	CSFL	-
CL	-	CFL	-	CSJL	-
SL	-	CJL	++	CSPL	-
FL	-	CPL	++	CFJL	++
JL	++	SFL	-	CFPL	++
PL	++	SJL	-	CJPL	++
		SPL	-	SFJL	-
		FJL	++	SFPL	-
		FPL	+	SJPL	+
		JPL	++	FJPL	++
				CSFJL	-
				CSFPL	+
				CSJPL	+
				CFJPL	++
				SFJPL	-
				CSFJPL	-

-, no pellicle; +, thin pellicle; ++, thick pellicle

Table S4. Summary of pellicle formation by different bacterial communities, including their presence and thickness (thick or thin).

Combination	Thickness
CG1	++
CG1G2	++
CG1G2G3	++
CG1G2G3G4	++
CG1G2G3G4G5	-

-, no pellicle; +, thin pellicle; ++, thick pellicle

C: CSFJPL
G1: *Pseudomonas prosekii*, *Pseudomonas iridis*
G2: *Pseudomonas* sp019345465, *Pseudomonas gessardii*
G3: *Pseudomonas weihenstephanensis*, *Pseudomonas kielensis*
G4: *Pseudomonas* brenneri, *Janthinobacterium* sp009208735
G5: *Pseudomonas* sp008369295, *Pedobacter antarcticus*

Table S5. The absence and presence of quorum sensing systems among 19 strains in the D-little hole community.

Community	Microorganisms	NCBI Accession #	QS								
			**CAI-1**			**AI-1**		**AI-2**		**AI-3**	
			**CqsAS**			**LuxIR**				**QseBC**	
			CqsA	CqsS	SprR	LuxI	LuxR	LuxQ	TqsA	QseC	QseB
D-Little hole	*Serratia liquefaciens*	SAMN57172073	0	0	1	0	0	0	1	1	4
	Carnobacterium maltaromaticum	SAMN42690019	0	0	0	0	0	0	1	0	0
	Pseudomonas prosekii	SAMN57172074	0	0	0	0	1	0	1	0	9
	Pseudomonas iridis	SAMN57172075	0	0	0	0	0	1	1	1	10
	Pseudomonas sp019345465	SAMN57172076	0	0	0	0	0	0	1	0	7
	Pseudomonas shahriarae	SAMN57172077	0	0	0	0	0	0	1	0	9
	Pseudomonas gessardii	SAMN57172078	0	0	0	1	0	0	1	0	8
	Pseudomonas weihenstephanensis	SAMN57172079	0	0	0	0	0	0	1	0	2
	Janthinobacterium tructae	SAMN57172080	1	1	0	0	0	0	1	0	9
	Pedobacter antarcticus	SAMN57172081	0	0	0	0	0	0		0	0
	Pseudomonas kielensis	SAMN57172082	0	0	0	0	0	0	1	0	2
	Pseudomonas brenneri	SAMN57172083	0	0	0	0	0	0	1	0	7
	Pseudomonas sp008369295	SAMN57172084	0	0	0	0	0	0	0	0	5
	Janthinobacterium sp009208735	SAMN57172085	1	1	0	0	0	0	1	1	4
	Flavobacterium spp.	SAMN57172086	0	0	0	0	0	0	0	0	0

[Footnote: The identification of amino acid sequences is over 40%, and the query coverage is over 60%. Homologous proteins with different origins of bacteria were combined and labelled as (multi)]. Genome accession numbers of the strain are provided in Table S4; protein accession numbers for the query sequences are provided in Table S5 [5–11].

Table S6. Relationship between bacteriocin, peptides molecules, and biofilm formation in three strains of *Carnobacterium maltaromaticum*.

Community	Strain ID	Bacteriocin[a)]	Relation to biofilm formation	References
Picnic 3 month (P3M)	*Carnobacterium maltaromaticum* FUA3987	Putative AgrB-like protein	transmembrane enzymes involved in the processing and secretion of autoinducing peptides (AIPs) in Gram-positive bacteria and *Listeria monocytogenes*	[12, 13]
		Piscicolin 126	QS regulates production of Piscicolin 126	[14, 15]
D-Little hole (DLH)	*Carnobacterium maltaromaticum* FUA3955	Sactipeptides	Anti-biofilm activity through disruption	[16]
		Carnobacteriocin BM1 (Carnobacterin B1)	QS regulates production of carnobacterin B1	[17, 18]
		Carnobacteriocin B2 (Carnocin CP52)	QS regulates production of carnobacteriocin B2	[17, 19]
		Carnolysin A1	Carnolysin is homologous to cytolysin and Carnobacteriocin X; when concentration of cytolysin increases biofilm formation increases; regulator of eDNA synthesis	[20]
		Piscicolin 126		
D-Drain in cooler (DDC)	*Carnobacterium maltaromaticum* FUA3957	Sactipepetides<br>Carnobacteriocin BM1 (Carnobacterin B1)<br>Carnobacteriocin B2 (Carnocin CP52)<br>Carnolysin A1<br>Carnolysin A2<br>Piscicolin 126		

Bacteriocins indicated in brackets are synonymous names for the same peptide sequence. Genome accession numbers of the strain are provided in Table S4; protein accession numbers for the query sequences are provided in Table S5.

Table S7. Protein screening of three-component regulatory system (CS-CbnK-CbnR) in three strains of *Carnobacterium maltaromaticum*.

Community	Strain ID	Protein	Relation to regulatory system	References
Picnic 3 month (P3M)	*Carnobacterium maltaromaticum* FUA3987	Histidine protein kinase (CbnK)	NA	[21, 22]
		Response regulator (CbnR)	NA	
D-Little hole (DLH)	*Carnobacterium maltaromaticum* FUA3955	Histidine protein kinase (CbnK)<br>Response regulator (CbnR)	utilize a phosphorylation cascade to transduce the CS signal into promoter activation	[10, 11]
D-Drain in cooler (DDC)	*Carnobacterium maltaromaticum* FUA3957	Histidine protein kinase (CbnK)<br>Response regulator (CbnR)	utilize a phosphorylation cascade to transduce the CS signal into promoter activation	[10, 11]

Protein accession numbers for the BlastP queries are provided in: https://doi.org/10.1128/jb.179.19.6163-6171.1997

References

1. Fugett E, Fortes E, Nnoka C. *Listeria monocytogenes* strain collection : development of standard *Listeria monocytogenes* strain sets for research. *J Food Prot* 2006;**69**:2929–2938.
2. Bohaychuk VM, Gensler GE, King RK, et al. Evaluation of detection methods for screening meat and poultry products for the presence of foodborne pathogens. *J Food Prot* 2005;**68**:2637–2647. https://doi.org/10.4315/0362-028X-68.12.2637
3. Van De Merwe C, Simpson DJ, Qiao N, et al. Is the persistence of *Listeria monocytogenes* in food processing facilities and its resistance to pathogen intervention linked to its phylogeny? *Appl Environ Microbiol* 2024;**90**:e00861. https://doi.org/10.1128/AEM.00861-24
4. Furrer B, Candrian U, Hoefelein C, et al. Detection and identification of *Listeria monocytogenes* in cooked sausage products and in milk by in vitro amplification of haemolysin gene fragments. *Journal of Applied Bacteriology* 1991;**70**. https://doi.org/10.1111/j.1365-2672.1991.tb02951.x
5. Parker CT, Russell R, Njoroge JW, et al. Genetic and mechanistic analyses of the periplasmic domain of the enterohemorrhagic *Escherichia coli* QseC histidine sensor kinase. *J Bacteriol* 2017;**199**. https://doi.org/10.1128/JB.00861-16
6. Khera R, Mehdipour AR, Bolla JR, et al. Cryo-EM structures of pentameric autoinducer-2 exporter from *Escherichia coli* reveal its transport mechanism. *EMBO J* 2022;**41**. https://doi.org/10.15252/embj.2021109990
7. Neiditch MB, Federle MJ, Miller ST, et al. Regulation of LuxPQ receptor activity by the quorum-sensing signal autoinducer-2. *Mol Cell* 2005;**18**. https://doi.org/10.1016/j.molcel.2005.04.020
8. Schaefer AL, Lappala CR, Morlen RP, et al. LuxR- and luxI-type quorum-sensing circuits are prevalent in members of the populus deltoides microbiome. *Appl Environ Microbiol* 2013;**79**. https://doi.org/10.1128/AEM.01417-13
9. Zaitseva Y V., Koksharova OA, Lipasova VA, et al. SprI/SprR Quorum sensing system of *Serratia proteamaculans* 94. *Biomed Res Int* 2019;**2019**. https://doi.org/10.1155/2019/3865780
10. Hornung C, Poehlein A, Haack FS, et al. The *Janthinobacterium* sp. HH01 genome encodes a homologue of the *V. cholerae* CqsA and *L. pneumophila* LqsA autoinducer synthases. *PLoS One* 2013;**8**. https://doi.org/10.1371/journal.pone.0055045
11. Shiratsuchi A, Sato S. Nucleotide sequence of trpE, anthranilate synthase I gene, of *Bacillus caldotenax*. *BBA - Gene Structure and Expression* 1991;**1090**. https://doi.org/10.1016/0167-4781(91)90201-V
12. Saenz HL, Augsburger V, Vuong C, et al. Inducible expression and cellular location of AgrB, a protein involved in the maturation of the staphylococcal quorum-sensing pheromone. *Arch Microbiol* 2000;**174**. https://doi.org/10.1007/s002030000223
13. Autret N, Raynaud C, Dubail I, et al. Identification of the agr locus of *Listeria monocytogenes*: Role in bacterial virulence. *Infect Immun* 2003;**71**. https://doi.org/10.1128/IAI.71.8.4463-4471.2003
14. Gursky LJ, Martin NI, Derksen DJ, et al. Production of piscicolin 126 by *Carnobacterium maltaromaticum* UAL26 is controlled by temperature and induction peptide concentration. *Arch Microbiol* 2006;**186**:317–325. https://doi.org/10.1007/s00203-006-0147-z
15. Jack RW, Wan J, Gordon J, et al. Characterization of the chemical and antimicrobial properties of piscicolin 126, a bacteriocin produced by *Carnobacterium piscicola* JG126.

Appl Environ Microbiol 1996;**62**:2897–2903. https://doi.org/10.1128/aem.62.8.2897-2903.1996
16. Roblin C, Chiumento S, Jacqueline C, et al. The multifunctional sactipeptide ruminococcin C1 displays potent antibacterial activity in vivo as well as other beneficial properties for human health. *Int J Mol Sci* 2021;**22**. https://doi.org/10.3390/ijms22063253
17. Quadri LEN, Sailer M, Roy KL, et al. Chemical and genetic characterization of bacteriocins produced by *Carnobacterium piscicola* LV17B. *J Biol Chem* 1994;**269**. https://doi.org/10.1016/s0021-9258(17)32702-3
18. Kleerebezem M, Kuipers OP, De Vos WM, et al. A two-component signal-transduction cascade in *Carnobacterium piscicola* LV17B: Two signaling peptides and one sensor-transmitter. *Peptides (NY)* 2001;**22**. https://doi.org/10.1016/S0196-9781(01)00494-6
19. Wang Y, Henz ME, Fregeau Gallagher NL, et al. Solution structure of carnobacteriocin B2 and implications for structure - Activity relationships among type IIa bacteriocins from lactic acid bacteria. *Biochemistry* 1999;**38**. https://doi.org/10.1021/bi991351x
20. Tulini FL, Lohans CT, Bordon KCF, et al. Purification and characterization of antimicrobial peptides from fish isolate Carnobacterium maltaromaticum C2: Carnobacteriocin X and carnolysins A1 and A2. *Int J Food Microbiol* 2014;**173**. https://doi.org/10.1016/j.ijfoodmicro.2013.12.019
21. Rohde BH, Quadri LEN. Functional characterization of a three-component regulatory system involved in quorum sensing-based regulation of peptide antibiotic production in Carnobacterium maltaromaticum. *BMC Microbiol* 2006;**6**. https://doi.org/10.1186/1471-2180-6-93
22. Quadri LEN, Kleerebezem M, Kuipers OP, et al. Characterization of a locus from *Carnobacterium piscicola* LV17B involved in bacteriocin production and immunity: Evidence for global inducer-mediated transcriptional regulation. *J Bacteriol* 1997;**179**. https://doi.org/10.1128/jb.179.19.6163-6171.1997
